# Systematic Review of the Long-Term Neuroimaging Correlates of Mild Traumatic Brain Injury and Repetitive Head Injuries

**DOI:** 10.3389/fneur.2021.726425

**Published:** 2021-09-30

**Authors:** Holly Victoria Echlin, Alma Rahimi, Magdalena Wojtowicz

**Affiliations:** Department of Psychology, York University, Toronto, ON, Canada

**Keywords:** mild traumatic brain injuries, repetitive head impact, neuroimaging, long-term, concussion, athletes, veterans, general population

## Abstract

**Objective:** To systematically review the literature on the long-term neuroimaging findings (≥10 years from exposure) for exposure in adulthood to mild traumatic brain injury (mTBI) and repetitive head impacts (RHIs) using neuroimaging across all available populations.

**Data sources:** Four electronic databases: MEDLINE, SPORTDiscus, PsycINFO, and EMBASE.

**Study selection:** All articles were original research and published in English. Studies examined adults with remote exposure to mTBI and/or RHIs from ten or more years ago in addition to any associated neuroimaging findings.

**Data extraction:** Parameters mainly included participants' population, age, years since head injury, race, sex, education level, and any neuroimaging findings. Scores for the level of evidence and risk of bias were calculated independently by two authors.

**Results:** 5,521 studies were reviewed, of which 34 met inclusion criteria and were included in this study. The majority of adults in these studies showed positive neuroimaging findings one or more decades following mTBI/RHI exposure. This was consistent across study populations (i.e., veterans, athletes, and the general population). There was evidence for altered protein deposition patterns, micro- and macro-structural, functional, neurochemical, and blood flow-related differences in the brain for those with remote mTBI/RHI exposure.

**Conclusion:** Findings from these studies suggest that past mTBI/RHI exposure may be associated with neuroimaging findings. However, given the methodological constraints related to relatively small sample sizes and the heterogeneity in injury types/exposure and imaging techniques used, conclusions drawn from this review are limited. Well-designed longitudinal studies with multimodal imaging and in-depth health and demographic information will be required to better understand the potential for having positive neuroimaging findings following remote mTBI/RHI.

## Introduction

Traumatic brain injuries (TBIs) are estimated to affect sixty-nine million individuals around the world each year (1) and according to the World Health Organization, mild traumatic brain injuries (mTBIs) account for approximately 70–90% of all treated TBI cases (2). MTBIs, some of which are termed concussions, refer to an alteration in brain function induced by biomechanical forces, such as an impact to the head or body, which results in a range of clinical signs and symptoms (i.e., physical, psychological, cognitive) (3). More recently, research has also been devoted to better understanding the long-term effects of repetitive head impacts (RHIs) which refers to milder impacts to the head or body which are associated with subthreshold or no clinical signs or symptoms upon impact (4). Given the prevalence of these injuries, there has been a growing interest in the potential long-term consequences of mTBI and cumulative effects of exposure to RHIs.

Prior to 2010, research was limited on this topic and generally focused on retired boxers (5) or specific case study investigations (6–9). Longitudinal data investigating the long-term sequelae of mTBI has been limited and only a few systematic reviews of the literature have focused on synthesizing such information beyond a decade injury exposure (10–17). These systematic reviews typically covered specific areas which either involved examining athletes, veterans, and the general population using one neuroimaging modality (e.g., diffusion tensor imaging; DTI) or using several neuroimaging modalities to examine only one population (e.g., athletes).

However, multimodal neuroimaging is critical for a comprehensive understanding of the potential long-term sequalae of mTBI/RHIs. Some studies using structural MRI have noted volume and thickness difference in individuals with a history of mTBI (18, 19). Studies using DTI to capture white matter integrity have noted evidence of diffuse axonal injury following injury. (20, 21) which may contribute to the disconnection and/or dysfunction of large-scale functional networks (22). In fact, altered functional connectivity has also been observed in asymptomatic individuals with a history of mTBI/concussion (23, 24). This highlights the importance of fMRI studies in understanding potential neural re-organization post-injury (25).

Additionally, assessing protein accumulation using PET scans can provide useful information regarding the neuropathological sequelae of exposure to mTBI/RHIs in the long term. That is, if an accelerated aging or neurodegenerative process is suspected, understanding protein deposition patterns can assist with disentangling various neuropathological processes and enabling identification and labeling of outcomes. Studies have also highlighted the utility of proton magnetic resonance spectroscopy (MRS) for its predictive value in quantifying metabolites (i.e., potential indicators of a persistent neuroinflammatory process) that may relate to post-injury recovery (26). These biomarkers may facilitate identifying injury or disease pathology (27, 28).

To date, a comprehensive systemic review of the neuroimaging literature across all populations with exposure to remote mTBI/RHIs has not been conducted. While prior systematic review articles have compared studies examining outcomes for long-term mTBIs, many of these focused on a single population (10, 16), or one particular method (e.g., only PET or DTI) (11–13). In addition, others examined evidence across the spectrum of traumatic brain injury severities (14, 15, 17), while the present study focuses on mTBIs. Furthermore, regular updates are required in a field that is burgeoning and quickly evolving in terms of scope and findings, particularly given the identified gap in the literature regarding the effects of mTBI beyond ten years post-injury (17). Therefore, with the aim of synthesizing findings about the long-term effect (i.e., ten or more years post-injury/exposure) of mTBI and RHIs on the brain, this systematic review will examine evidence from a broad range of neuroimaging modalities to provide a comprehensive, and in-depth discussion the current state of the literature.

## Methods

### Search Strategy

This study was developed in accordance with PRISMA guidelines. Initial keywords were selected based on a previous relevant review (10) and from scans of the relevant literature (i.e., snowball searching). These keywords were then submitted to a librarian with expertise in health studies and systematic reviews in order to collaboratively draft a MEDLINE search strategy. The draft search strategy was adapted in accordance with the CADTH Peer Review Checklist for Search Strategies according to the Press 2015 Guideline Statement. Afterwards, this draft was tested to ensure that known relevant studies were included. The resulting MEDLINE search was translated to conform to each of the other databases used (see Supplementary Table 1 for the search strategy for MEDLINE). The following databases were comprehensively searched by investigator HE: MEDLINE, SPORTDiscus, PsycINFO, and EMBASE. Gray literatures searches were also performed (e.g., snowball searches, google searches, and ProQuest Dissertations and Theses search). The search was conducted on January 12, 2021 and was limited to articles that were published in English, with no date restrictions.

### Selection Criteria

The selection criteria for inclusion included: (1) the samples included at least one group of adults who had a history of RHIs or at least one mTBI (i.e., complicated or uncomplicated), (2) a follow-up period of at least ten years post-injury, (3) neuroimaging acquired >10 years after exposure to mTBI/RHI, (4) a minimum average age of 28 (i.e., studies were searched to ascertain that the average age for the reported injuries was 18 years or older, to identify injuries that occurred in adulthood) (5) discussion of outcomes and sequelae due to head injury (e.g., seizures, smaller brain volumes, etc.), (6) the availability of the full text in a journal or database, and (7) the use original research that has been published in English. Studies were excluded from the review if (1) they were animal studies, review articles, case reports, book chapters, conference abstracts, editorials/commentaries/expert opinion, theses, or dissertations, (2) individuals were diagnosed with non-traumatic brain injuries (e.g., stroke or epilepsy), (3) the ten-year follow-up evaluation occurred before adulthood (e.g., before age 18), and (4) the sample included only clinical populations with other neurological diagnoses. However, studies with clinical populations could only be included if the clinical population was compared separately or used as a control group for comparison with an mTBI/RHI-exposed sample that met inclusion criteria. Studies were also included if rationale could be provided to account for the ten years between injury and neuroimaging (e.g., professional athletic career ended more than ten years ago, according to age at neuroimaging acquisition) even when participants' time since last injury was not explicitly reported.

Two of the authors (HE and AR) discerned the studies' eligibility for inclusion by first independently reviewing titles and abstracts. Studies that did not meet the exclusion criteria at this step were further evaluated in the full text review stage. Article inclusion was decided upon via discussion between both authors.

### Risk of Bias and Level of Evidence

The level of evidence and risk of bias of the included studies were rated by two authors, and disagreements in ratings were settled by discussion between the two. The Downs and Black checklist was used to assess the risk of bias (29), while the level of evidence was assessed using the Oxford 11 Level of Evidence (30). While the provided cut-offs for scores embedded in the Downs and Black checklist indicate that scores lower than 14 suggest poor methodological quality (31), applying this criteria to observational studies that do not include randomization, blinding, and/or reporting of adverse events, etc., results in lower scores. However, given that others have used this measure in this field (10) and have reported similar ratings, the broad utility of this measure for the current review is to provide a way to compare the relative risk of bias between studies within this paper. Similarly for the Oxford 11 Level of Evidence rating, to understand the integrity of the design of one paper, one might check the level of evidence score in order to compare it with a similar study or against the average provided (see section 3).

### Data Extraction

After the abstracts and titles were screened and the full texts were reviewed by authors, HE and AR, a standardized method in Covidence was used to extract the data from the included articles. One author extracted the data and a second author reviewed it for accuracy and to confirm that it was comprehensive. Once the data was extracted, all three authors reviewed the finalized tables. The information extracted from the included studies mainly consisted of study design, demographic information about the sample, the type of head injury exposure and the context within which it occurred, the type of neuroimaging modality used for assessment, any reports of potential diagnoses, and time since the injury. This systematic review was registered with the International Prospective Register of Systematic Reviews (PROSPERO; registration number CRD42020178475).

## Results

**Figure 1:**
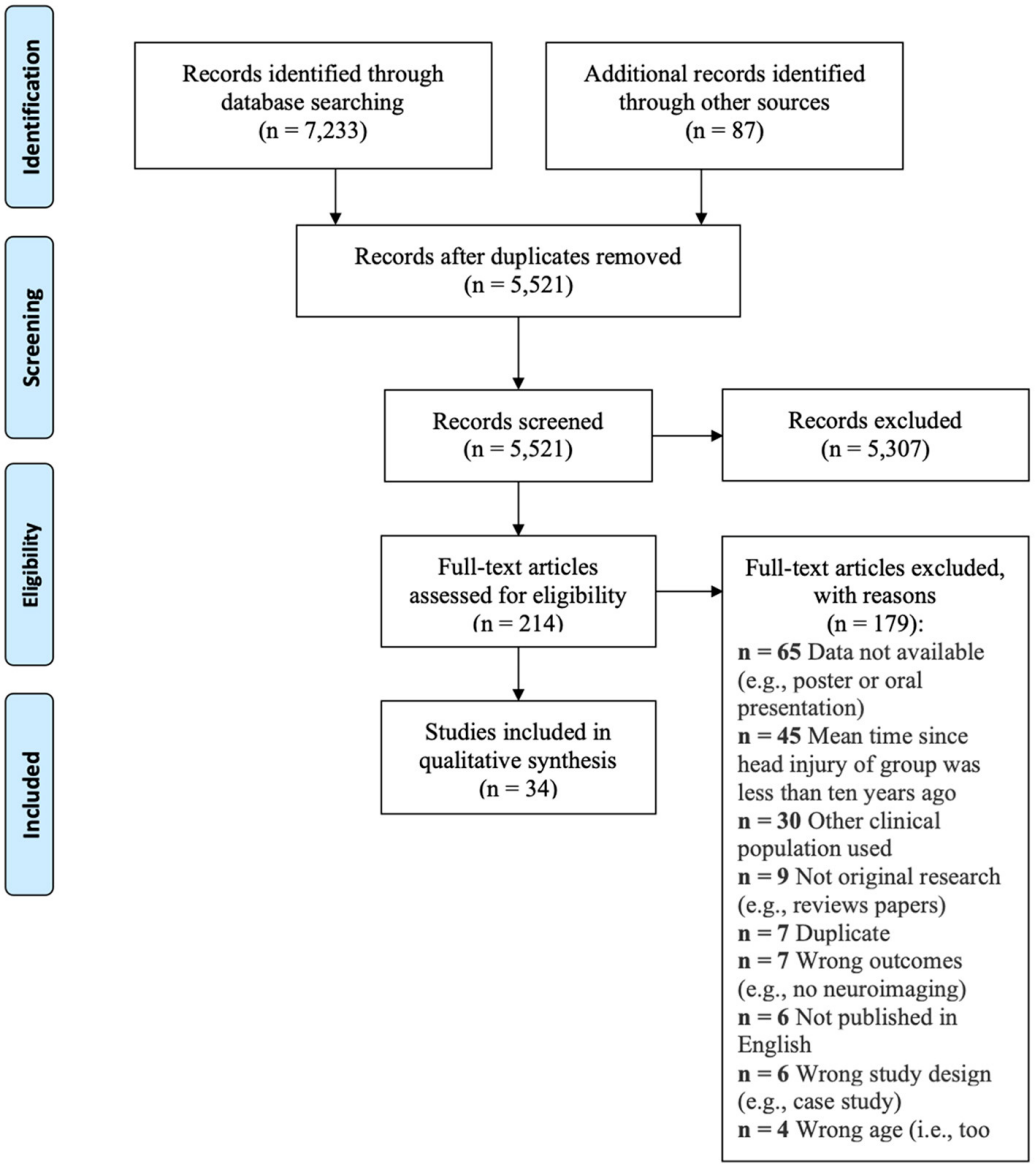
PRISMA flow diagram.

In total, the database searches and the gray literature searches resulted in 7,233 articles being identified (see Figure 1). Covidence identified and removed 1,712 duplicates. In the first stage of screening, 5,521 articles were reviewed. After exclusion of ineligible articles, 214 articles were screened in the full-text review stage and 34 were deemed eligible for inclusion in this review. The online Supplementary Table 2 contains relevant information from each of the studies included in this review. Online Supplementary Table 3 includes a summary of the risk of bias ratings and ratings of the methodological quality of included studies. According to the Downs and Black criteria for rating methodological quality, the average score for the included studies was 9.8. Given the context of the studies that were included as well as the guidelines for their assessment, the methodological quality of the studies in this review overall are considered poor. However, it is important to note that ideal control groups are difficult to establish for this population and that certain methodological procedures (e.g., blinding) are not possible for observational studies, which lowered the scores. Two studies in this review were rated a level of 3 (i.e., cohort study) for their level of evidence. The remaining 32 studies were rated as a level 4 (i.e., case-series).

### Description of Studies

There were 34 articles identified that characterized the brain and the potential long-term associations of mTBIs and/or RHIs using neuroimaging. The imaging techniques used in these papers included diffusion tensor imaging (DTI; *n* = 12 studies), measures of functional connectivity (i.e., functional MRI or magnetoencephalography; *n* = 9 studies), magnetic resonance spectroscopy (MRS; *n* = 5 studies), optical coherence tomography (OCT; *n* = 2 studies), perfusion weighted imaging (PWI; *n* = 1), arterial spin labeling (ASL; *n* = 1), positron emission tomography (PET; *n* = 4 studies), structural neuroimaging measures (e.g., MRI scans; *n* = 18 studies), and susceptibility weighted imaging (SWI; *n* = 3 studies). The populations examined included veterans, athletes, and individuals from the general population who had sustained a mTBI or were exposed to RHIs. The majority of studies examined professional or university-level athletes (*n* = 26; American football was the most common sport), and four of these articles included professional fighters. Only two studies used a veteran population, five included the general population, and one study had a mix of athletes and the general population. In total, two of the included studies were longitudinal and the rest were cross-sectional. Almost all of the included studies reported significant differences between groups with regard to brain structure, function, and pathology.

### Definition of mTBIs/Concussion and Repetitive Head Injuries

MTBI/concussion were typically defined using recommended guidelines that were relevant when the paper was published. In several studies, participants were asked to self-report prior diagnoses of mTBIs, though the description of how a mTBI/concussion was defined was often not fully transparent (32–37). In several studies, participants were asked to self-report their concussion history after being provided with a standard definition (3, 38–43) according to guidelines that were most up-to-date at the time of study publication (44–58). Other studies used medical chart reviews (59) or questionnaires (60) and/or interviews to attain this data, with some studies also using certified neuropsychologists or physicians to collect such information (48, 49, 61–63). For participants who were medically diagnosed with mTBI, the Glascow Coma Scale was often used to ascertain that participants' scores fell between 13 to 15. Of the studies that reported on participants' experience of loss of consciousness (LOC) or post-traumatic amnesia (PTA), injury severity was verified by participants experiencing LOC for 30 minutes or less, and/or having less that 24 hours of PTA, based on criteria from the Mild Traumatic Brain Inury Committee (64).

Definitions and operationalization of RHIs were variable. Studies opted to define RHIs in multiple ways, typically using estimation methods. For instance, some studies used exposure to contact sports as a proxy for repetitive head impact exposure (14, 34, 65–69) which sometimes included participants' estimates of ball headings per session/week (70, 71) and/or the number of times players recalled being taken out of play due to head impact (72).

### Diffusion Tensor Imaging

Several studies captured in this review examined white matter pathology in retired professional athletes (*n* = 8). Some studies reported differences in fractional anisotropy (FA) between retired professional players and controls. For instance, Hart et al. observed that retired players with cognitive or mood-related symptoms had lower FA in the corpus callosum, bilateral frontal and parietal regions, and the left temporal lobe than matched controls (51). However, athletes without mood or cognitive symptoms in this study were not significantly different from controls in terms of white matter FA. In addition, Zivadinov et al. reported no observable differences between retired professional athletes (NFL and NHL) and controls, and no associations between lifetime concussion history and white matter disruption (69). In former college-level football athletes associations were observed between white matter disruption (i.e., lower FA) in the corpus callosum and superior longitudinal fasciculus, and greater number of concussion as well as longer career duration (32). In contrast, greater FA was observed in the retired NFL players in this study. It was also observed that retired NFL and college players in non-speeded positions (i.e., offensive or defensive linemen) with a higher number of concussions had lower FA in the forceps minor compared to those with zero or one reported concussion.

Two studies examined retired CFL players for associations between RHIs or mTBI exposure and indicators of white matter pathology. Multani and colleagues found higher AD (but not FA, MD, or RD) in the right hemispheres of the retired CFL players, specifically in the superior longitudinal fasciculus, corticospinal tract, and anterior thalamic radiations (58). Using machine learning, Goswami et al. found that MD and RD (but not FA) in the uncinate fasciculus (particularly at the orbitofrontal and temoporal ends) differentiated controls and athletes with 79–84% sensitivity and specificity (45). In retired soccer players, a higher reported frequency of heading the ball (more than 885 per year) was associated with lower FA at three locations in the temporo-occipital white matter in a group of amateur soccer players (71). In addition, this study did not find observable differences between professional athletes and controls or associations with lifetime concussion history when looking at white matter disruption.

For boxers, one study found no group differences in FA or the apparent diffusion coefficient (ADC) (68), while another study using the same sample did, but in different regions (67). Lower FA in the splenium and higher MD in the genu of the corpus callosum was found for boxers (67). Additionally, years of boxing was associated with lower FA in the right ventral striatum, right uncinate fasciculus, and right cerebral peduncle and with the ADC in the left inferior longitudinal fasciculus and left uncinate fasciculus (68).

Studies that examined adults in the general population who were exposed to remote mTBI typically found higher AD, RD, and MD (but not FA) in areas that included the fornix/stria terminalis, anterior corona radiata, superior longitudinal fascilus, cingulum angular bundle, the anterior aspect of the corpus callosum, and frontal white matter (35, 49, 61). These studies demonstrated white matter differences in adults with remote mTBI compared to healthy controls who were either the same age or younger. In contrast, Rajesh et al. found no differences in FA between adults with remote mTBI exposure and controls (46). See Table 1 for a summary of the DTI findings.

**Table 1:** Aggregated results for all DTI studies.

**Lead author**	**FA**	**MD/ADC**	**RD**	**AD**
Hart et al. (51)	Corpus callosum (↓) Bilateral frontal regions (↓) Bilateral parietal regions (↓) Left temporal lobe (↓)	N/R	N/R	N/R
Zivadinov et al. (69)	Null findings for group differences	Null findings for group differences	N/R	N/R
Clark et al. (32)	Corpus callosum (↓ for college players but ↑ for retired players) Superior longitudinal fasciculus (↓ for college players but ↑ for retired players)	N/R	N/R	N/R
Multani et al. (58)	Null findings for group differences	Null findings for group differences	Null findings for group differences	Superior longitudinal fasciculus (↑) Corticospinal tract (↑) Anterior thalamic radiations (↑)
Goswami et al. (45)	N/R	Able to differentiate between controls and athletes (79-84% sensitivity and specificity)	Able to differentiate between controls and athletes (79-84% sensitivity and specificity)	N/R
Lipton et al. (71)	Temporo-occipital white matter (↓)	N/R	N/R	N/R
Wilde et al. (68)	Null findings for group differences Right ventral striatum (↓; with years of boxing) Right uncinate fasciculus (↓; with years of boxing) Right cerebral peduncle (↓; with years of boxing)	Null findings for group differences Left inferior longitudinal fasciculus (↑; with years of boxing) Left uncinate fasciculus (↑; with years of boxing)	N/R	N/R
Ware et al. (67)	Splenium of the corpus callosum (↓)	Genu of the corpus callosum (↑)	Null findings for group differences	N/R
June et al. (35)	N/R	N/R	Fornix/stria terminalis (↑)	Anterior corona radiata (↑) Superior longitudinal fasciculus (↑)
Tremblay et al. (49)	N/R	Anterior corpus callosum (↑) Frontal white matter (↑)	N/R	N/R
Chong et al. (61)	N/R	Left cingulum angular bundle (↓) Right cingulum angular bundle (↑) Left corticospinal tract (↑) Right inferior longitudinal fasciculus (↓) Uncinate fasciculi (↓) Forceps major (↑) Forceps minor (↓)	Anterior thalamic radiations (↓) Left and right cingulum angular bundle (↑) Left and right cingulum cingulate gyrus (↑) Left and right corticospinal tract (↑) Left uncinate fasciculus (↓) Right uncinate fasciculus (↓) Forceps minor (↓)	N/R
Rajesh et al. (46)	Null findings for group differences	N/R	N/R	N/R

*Directionality, depicted using arrows, is reported for the experimental group in reference to control groups unless otherwise specified. N/R, not reported*.

### Functional Connectivity Using MRI and MEG

#### Task-Based fMRI

Several studies used event-related fMRI to examine blood oxygen level dependent (BOLD) signal changes while participants performed cognitive tasks (*n* = 4). Two studies used item/relational memory tasks (33, 57), when comparing middle-aged adults with remote mTBIs vs. younger adults with recent mTBIs, Monti et al. found that the younger adults showed more activity in the prefrontal cortex and parietal regions than the middle-aged adults overall (57). Also, the middle-aged adults with remote mTBI had less neural activity compared to matched controls in several regions in the right medial prefrontal cortex, bilateral frontopolar cortex, and middle and superior frontal gyri during memory performance (i.e., areas that may be associated with better performance). Using retired NFL players with low vs. high frequency concussion exposure (three or more concussions), Ford and colleagues found that both concussion groups recruited similar regions for item memory retrieval, but differences in neural recruitment were notable during relational memory retrieval (33). Specifically, the high frequency concussion group showed less efficiency in neural recruitment and tended to recruit medial PFC regions more than the low frequency concussion group.

Two studies examined aspects of executive functioning and working memory (32, 72). Retired NFL players who were given the executive functioning task showed hyperactivation and hypoconnectivity of the dorsolateral frontal and frontopolar cortices and tended to recruit frontopolar regions when task difficulty increased, while controls did not (72). For retired NFL players who were given the working memory task, no association between concussion history or career duration were found with BOLD signal. However, players in non-speeded positions (offensive or defensive linemen) with three or more concussions had lower BOLD signal changes during a working memory task than the low frequency concussion group. The opposite pattern was found for speed position athletes, such that lower BOLD signals were found in the low frequency concussion group (32).

#### Resting-State fMRI

Two studies used resting-state functional MRI (rs-fMRI) to examine group differences between adults with mTBI history and controls. Goswami and colleagues found higher rs-fMRI connectivity between the anterior temporal lobe and orbitofrontal cortex in retired CFL players compared to controls (45). In another study, decreased functional connectivity between a key node of the default mode network (i.e., the posterior cingulate cortex) and the right frontal pole/anterior prefrontal cortex was observed in only the younger adults with recent mTBIs compared to controls but not in the older adults with remote mTBIs and controls (46).

One study used resting-state magnetoencephalography, to examine small-worldness, clustering coefficient, and modularity in veterans who sustained a mTBI during their deployment approximately ten years ago (47). They found that those who had developed PTSD showed higher levels of small-worldness and clustering coefficient than the group who did not develop PTSD. There were no controls included in this study.

### Magnetic Resonance Spectroscopy

De Beaumont et al. reported glutamate/H_2_O ratios in the M1 region negatively correlated with the number of reported concussions in retired former athletes (62). An age by group interaction was observed for M1 glutamate concentrations indicating further decreases in M1 glutamate with age for those with a head injury history. Another study quantified metabolites in 15 retired athletes (who sustained head injuries several decades ago) in the medial temporal lobes and the prefrontal cortices including: N-acetylaspartate (NAA), myo-inositol (mI), choline-containing compounds (Cho), as well as H_2_O for an internal reference (48). They found significantly increased ratios of mI/H_2_O and abnormal reductions in Cho in the left medial temporal lobes and increased Cho when referenced to H_2_O, in the right prefrontal cortex of retired athletes.

In a sample of retired professional soccer players with no history of TBI but exposure to RHIs from ball headings, notable differences in neurochemistry were identified in comparison with non-contact sport athlete controls (73). Cho and mI were significantly elevated in the soccer group and mI and glutothione were significantly related to lifetime estimates of RHIs in the soccer group. In contrast, Zivadinov et al. reported that they found no long-term differences in relative concentrations of NAA, glutamine (Glu), and glutamate (Gln), relative to the concentration of creatine (Cr) and phosphocreatine (PCr) in the corpus callosum and fornix between retired NHL/NFL players and non-contact sport controls (69).

Finally, using an in vivo method of visualizing atomic structures/concentrations in 2D for differentiating checmicals called Localized COrrelated SpectroscopY (L-COZY), Lin and colleagues examined five retired professional athletes (football, wrestling, and baseball) and five matched athlete controls and found significantly higher levels of Gln/Glu (31%), choline (65%), fucosylated molecules (60%) and phenylalanine (46%) in the former athlete group (66).

### Cerebral Blood Flow and Volume

Using perfusion weighted imaging (PWI), Zivadinov and colleagues reported no significant differences in cerebral blood volume (CBV), perfusion cerebral blood flow (CBF), and mean transit time (MTT) in white matter-signal abnormalities, global and regional gray matter, and white matter brain structures between retired NHL/NFL players and non-contact sport controls (69). In contrast, Hart et al. used arterial spin labeling (ASL) to compare retired NFL players with cognitive impairments and/or depression to retired NFL players without such impairments and matched controls (51). Retired NFL players with cognitive/mood impairments had decreased bloodflow to the left temporal pole and increased bloodflow to the superior temporal gyrus and inferior parietal lobule than both control groups. Altered bloodflow to these areas was associated with neurocognitive performance (e.g., poorer naming, verbal memory, and word finding ability).

### Positron Emission Tomography

Small et al. examined PET scans after intravenous injections of 2-(1-{6-[(2-[F-18]fluoro-ethyl)(methyl) amino]−2 naphthyl}ethylidene)malononitrile (FDDNP) to measure tau and amyloid in a small sample of retired NFL players with mood and cognitive symptoms (*n* = 5) compared to matched controls (36). They found that FDDNP binding signals were higher in the retired NFL group than controls in the caudate, putamen, thalamus, subthalamus, midbrain, cerebellar white matter, and the amygdala. A recent PET tau imaging study used 5mCi of [F-18]AV-1451 tracer in a group of retired professional and semi-professional contact sport athletes who were split into groups depending on whether they were carriers of one/two copies of an APOE4 allele (50). They found that APOE4 carriers had significantly higher cortical gray matter PET tau SUVR values, suggesting higher susceptiblity to increased cortical protein deposition for this group.

In a non-athlete study, subjects who were involved in the Alzheimer's Disease Neuroimaging Initiative (ADNI) were split into groups based on whether they had been diagnosed with AD, had mild cognitive impairments (MCI) due to AD, had preclinical AD, or were healthy controls (37). In those with preclinical AD and self-reported mTBI exposure, average cortical thickness was smaller when collapsing across the AD-vulnerable regions and this difference was specifically observed in the precuneus and superior and inferior parietal cortices, compared to non-mTBI controls. The average cortical thickness of these eight regions was also correlated with CSF T- Tau in the preclinical mTBI group. In addition, a longitudinal study that used 15O-water PET, found higher resting cerebral blood flow in the orbitofrontal and lateral temporal regions in adults from the general population with exposure to mTBI (35). Both increases and decreases were seen in prefrontal, cingulate, insular, hippocampal, and ventral temporal regions with longitudinal follow-up.

### Spectral Domain Optical Coherence Tomography (OCT)

Kelman and colleagues used OCT (i.e., a potential indicator of white matter atrophy) to measure thinning of the retinal nerve fiber layer (RNFL) in a sample of 13 retired rugby league players in comparison with normative data (52). The RNFL was four micrometers thinner in the athlete group than the comparison sample. The specific areas that were significantly different between the retired athletes and normative data were the left inferonasal and left nasal sectors. This technique was also used in a longitudinal study that examined these outcomes in a veteran population (63). They reported that veterans with a history of mTBI had greater RNFL thinning compared to controls and that greater tissue loss was associated with higher ratings of mTBI severity (i.e., using Minnesota Blast Exposure Screening Tool severity scores).

### Structural Imaging

#### Cavum Septum Pellucidum (CSP)

Several studies examined the occurrence and characteristics of a cavum septum pellucidum. Using a sample of 50 amateur boxers with high match (i.e., higher number of matches; average of 54 fights in their career) vs. 25 low match exposure (i.e., average of 6 fights in their career) to examine whether morphological changes could be found when compared to track and field/soccer player controls (34). Using a 0.5T MRI, they reported no differences between these groups in the width of the ventricular system, anterior horn index, width of cortical sulci, signs of vermian atrophy, or the occurrence of a CSP. However, they observed that a CSP was found more often in track and field athlete controls than the boxers. Using a 1.5T MRI, Casson et al. found that 7% of their sample of retired NFL players had evidence of brain atrophy (i.e., enlarged ventricles and thinned corpus callosums) and large CSP, while 69% had a small cavum septum pellucidum (44).

More recently, 3T MRI was used to examine the occurrence and length of CSP, as well as its ratio to septum length in 72 former NFL players (presenting with cognitive, mood, and behavioral symptoms) and 14 former non-contact sport athlete controls (53). The authors found that former symptomatic NFL players had a higher rate of CSP as well as greater length of CSP (including its ratio to septum length). The presence of a CSP was also more common in a group of retired NFL players with cognitive and mental health symptoms (94%) than matched controls (18%) (59), and the length of the CSP in retired NFL players was significantly longer as well. Similar to these findings, another sample of 45 retired NFL players found that 7% had a CSP, 71% had a small one, and 22% did not have one (74).

#### Subcortical Structures

Studies that focused on examining differences between groups in subcortical volumes typically examined the hippocampus, amygdala, corpus callosum, and cingulate gyrus in retired professional athletes. Most recently, Bryant et al. examined structural MRI scans in a sample of older retired professional fighters and younger active professional fighters to compare brain volumes and correlations with age at first exposure (AFE) to fighting (65). Earlier AFE was associated with smaller volumes in the posterior corpus callosum and bilateral hippocampi and amygdalae in both groups. In the active fighters' group, there was also an association between AFE and smaller left amygdala volume while smaller right amygdala volume was associated with earlier AFE in the retired fighters group.

Studies consistently identified atrophy in the hippocampus (35, 56, 57) and less consistently in other structures like the amygdala or cingulate gyrus in those with remote mTBI exposure (55). For instance, in a comprehensive longitudinal study, (35) adults with mTBI history had smaller volumes than controls in white matter in the temporal lobe and the hippocampus at baseline, but volumes remained stable across subsequent visits. In retired CFL players, the number of years in the CFL was also significantly related to smaller volumes in the bilateral hippocampus and amygdala (56).

#### Cortical Thickness

For studies that examined cortical thickness in adults with exposure to remote mTBI, some consistent patterns of reduced thickness have been observed. Thinner temporal lobes (37, 45, 48, 70), frontal lobes (48, 70), and areas of the parietal lobes (37, 48, 70) have been noted in retired CFL players, former professional soccer players, adults with mTBI history and preclincal AD, and former college/university-level athletes who played ice hockey or football.

Tremblay and colleagues found that for former university/college athletes, increased age and having a mTBI history was associated with greater lateral ventricular volume expansion and thinner cortices (i.e., frontal, temporal, and parietal regions) compared to age matched controls (48). Additionally, former professional soccer players who had long-term exposure to heading the ball (i.e., played the sport for an average of 22.4 years) were found to have more cortical thinning with age in the occipital cortex (i.e., in addition to the temporal, posterior parietal, and left frontal cortices) (70).

Finally, adults from the general population with mTBI exposure and preclinical AD (described in section Cerebral Blood Flow and Volume) had reduced cortical thickness than controls when averaged across eight AD-vulnerable regions (i.e., superior and inferior parietal cortex, middle and inferior temporal gyrus, precuneus, posterior cingulate cortex, entorhinal cortex, and temporal pole), and in three of these regions specifically, including the precuneus and superior and inferior parietal cortex (37). Of note, no group differences were found in any of these regions between controls and adults with mTBI and normal cognition.

Similarly, a recent study examining cortical thickness in a sample of older adults with remote mTBIs compared to controls found no significant differences between groups as well (46). See Table 2 for a summary of the structural imaging findings.

**Table 2:** Aggregated results for all structural imaging studies.

**Lead author**	**Cavum Septum Pellucidum (CSP)**	**Subcortical**	**Cortical**
Haglund et al. (34)	Null findings for group differences	Null findings for group differences (i.e., vermis)	Null findings for group differences (i.e., cortical sulci)
Casson et al. (44)	CSP (↑)	Corpus callosum (↓)	N/R
Koerte, et al. (53)	CSP (↑)	N/R	N/R
Gardner et al. (59)	CSP (↑)	N/R	N/R
Kuhn et al. (54)	CSP (↑)	N/R	N/R
Bryant et al. (65)	N/R	Posterior corpus callosum (↓; with years of fighting exposure) Bilateral hippocampi (↓; with years of fighting exposure) Bilateral amygdalae (↓; with years of fighting exposure)	N/R
June et al. (35)	N/R	Temporal lobe white matter (↓) Bilateral hippocampi (↓)	N/R
Misquitta et al. (56)	N/R	Bilateral hippocampi (↓)	N/R
Monti et al. (57)	N/R	Bilateral hippocampi (↓) Null findings for group differences in the putamen, caudate, and thalamus	N/R
Lepage et al. (55)	N/R	Bilateral hippocampi (↓) Bilateral amygdalae (↓) Bilateral cingulate gyri (↓)	N/R
Wang et al. (37)	N/R	N/R	Null findings for normal cognition group Inferior parietal cortex (↓) Superior parietal cortex (↓) Precuneus (↓) Mean thickness of 8 cortical regions (↓)^a^
Goswami et al. (45)	N/R	N/R	Anterior temporal lobe (↓)
Tremblay et al. (48)	N/R	N/R	Bilateral frontal cortex (↓) Right temporo-parietal cortex (↓) Right temporal cortex (↓)
Koerte, et al. (70)	N/R	N/R	Bilateral temporal cortex (↓) Bilateral posterior parietal cortex (↓) Bilateral occipital cortex (↓) Left frontal cortex (↓)
Rajesh et al. (46)	N/R	N/R	Null findings for group differences

*Directionality, depicted using arrows, is reported for the experimental group in reference to control groups unless otherwise specified. N/R, not reported.*

a *The 8 cortical regions include the entorhinal cortex, temporal pole, inferior temporal gyrus, middle temporal gyrus, inferior parietal cortex, superior parietal cortex, precuneus, and posterior cingulate cortex*.

#### Ventricular and Global Brain Volume

Two studies examined ventricular size, skull diameter, and global brain volume. Zivadinov and colleagues found no significant differences between retired NHL/NFL players and non-contact sport controls when examining global and regional brain volumes (69). Another study examining professional fighters and controls however, reported that the maximum width of the anterior horns of the lateral ventricles and the interior diameter of the skull were significantly larger in the professional fighters (68).

### Susceptibility Weighted Imaging

Three studies used SWI to detect hemosiderin deposition, which may be indicative of prior hemorrhages. Two studies examined the same sample of 45 retired NFL players and found that 9% of participants had microbleeds in the parenchyma of the brain (44, 54). In contrast, no significant differences in the presence of cerebral microbleeds were observed between retired professional contact sport athletes and noncontact sport athlete controls in another study, though the control group had a higher number of participants with at least one cerebral microbleed (i.e., 33%) than the retired athletes group (i.e., 9.5%) (69).

## Discussion

**Figure 2:**
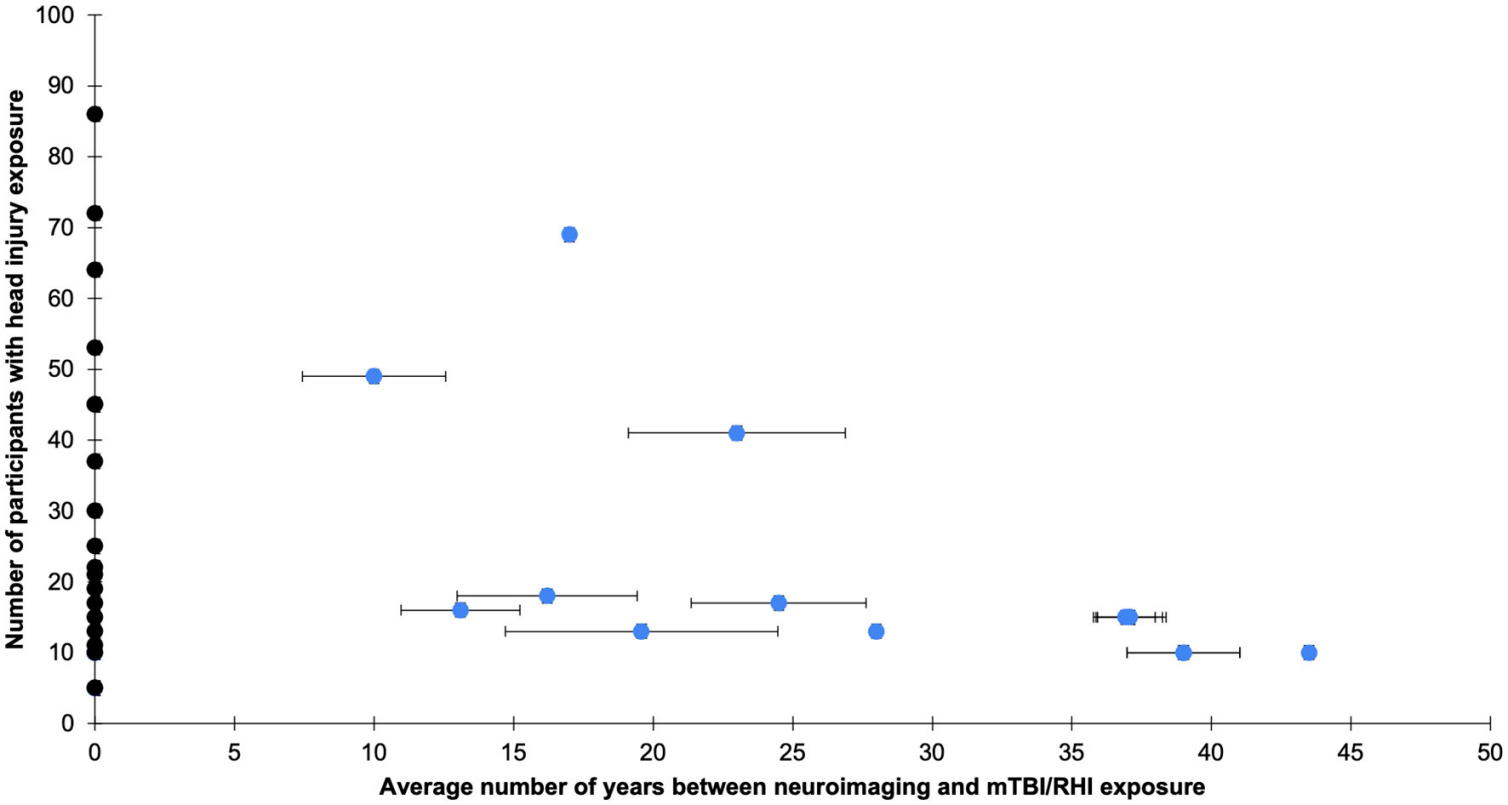
The average years since head injury exposure and number of participants with head injury exposure per study. Bars indicate the standard error and were plotted where available. Black dots represent studies that did not explicitly state years since head injury exposure but used other estimates, such as years since playing sport, or met criteria because follow-up occurred at least one decade after exposure to sport. See Supplementary Tables 1–3 for more details.

A total of 34 studies were identified that related to the long-term effects of mTBIs and RHIs on the brain (see Figure 2). The included studies used various neuroimaging techniques (i.e., PET scans, MRS, PWI, ASL, MTI, DTI, OCT, SWI, or fMRI) to examine brain health. All of the studies included in this review had a lower level of evidence (i.e., level four), with the exception of two studies that were rated a level three. In addition, risk of bias scores were below the cut-off value of 14 for most of the included studies which indicates a threat to their internal and external validity.

### Athletes

The majority of the neuroimaging studies included in this review were conducted with retired professional athletes. Of these, the majority of the samples were comprised of retired NFL or CFL players (32, 33, 36, 44, 45, 50, 51, 53, 55, 56, 58, 59, 72, 74, 75). The other studies included professional fighters (34, 65, 67, 68) or athletes from other contact sports (48, 49, 52, 62, 66, 69–71, 73). The neuroimaging research done in former NFL or CFL players has revealed differences compared to control samples in the presence and greater length of cavum septum pellucidum and volumes in the amygdala and hippocampus using structural MRI (53, 55, 56, 59). There was some evidence of differences in white matter microstructure using DTI, most consistently observed in the corpus callosum and superior longitudinal fasciculus (32, 51, 58), however, not all studies reported all DTI metrics (i.e., FA, RD, MD, AD) and/or similar regions of observed differences, if any (see Table 1). There was also some evidence of altered neural recruitment in areas supporting relational memory performance as identified by fMRI (33, 57), and greater accumulations of amyloid-β and neurofibrillary tangles shown on PET scans (36, 50). Though studies were cross-sectional and correlational, findings suggested a relationship between exposure to remote mTBI and/or RHIs and observable differences in brain structure and function using neuroimaging. However, it is notable that there was some variability in reported differences (e.g., observed differences in different regions and/or using different metrics, or differences between studies in the brain regions that they chose to investigate) and that there was limited replication of findings. Further, there was significant variability in the operationalization of mTBI and RHIs, making comparisons across studies difficult.

Findings from studies that examined former professional fighters were conflicting in that some found no brain-related differences between fighters and controls (34, 68), while others did (67). In the earlier study, it is noteworthy that the use of lower-strength magnet (0.5 tesla) might have contributed to some variable findings, in addition to making comparisons to the more recent studies (i.e., that use higher field strength MRI) difficult. Overall, two studies found evidence of similar white matter disruptions in the corpus callosum associated with the duration of exposure to boxing in professional fighters (65, 67). Future studies should aim to replicate findings about the long-term effects of RHIs in this population, specifically by examining the uncinate fasciculus and inferior longitudinal fasciculus.

Other studies examined athletes who played a variety of professional contact sports including rugby (52), soccer (70, 71, 73), a mix of several sports (66, 69) and/or levels of play (e.g., university level and professional) (49), as well as university-level hockey/football (48, 62). The imaging techniques used in these studies were also diverse. However, there was evidence of different levels of metabolites in several brain regions for athletes compared to controls which may be indicative of neuroinflammation (48, 62, 66, 73) and white matter disruption in widespread areas (49, 52, 71). As per the above sections, methodological considerations (i.e., distinct study designs and methods for selecting brain regions and quantifying metabolites) limit the conclusions that can be drawn from these data.

### Veterans

Published articles that focused on the long-term neuroimaging findings associated with RHIs/mTBI in veterans were limited. With only two studies identified in relation to this topic, the ability to make interpretations was severely limited. One study reported thinning of retinal nerve fiber layer (RNFL) thickness (63) and the other found altered functional connectivity (i.e., hyperconnectivity) after mTBI was associated with post-deployment development of PTSD (47). More research is needed to understand whether RNFL thickness is truly an indicator of white matter loss and neurodegeneration in this population. Also, given that hyperconnectivity was only seen in those who had developed PTSD, future research will be needed in order to confirm group differences and to better understand preliminary findings about functional connectivity in those who do and do not develop PTSD. Overall, a greater amount of research is needed to better understand the long-term consequences of RHIs and mTBI in veteran populations.

### General Population

Six of the studies that were included in this review examined participants from the general population. For two of these studies, groups were divided into younger and older adults who had recent or remote mTBIs, respectively for comparisons of brain structure and functional activation (46, 57). Both of these studies reported hypoactivation in several brain areas along with greater white matter disruption and smaller cortical and subcortical volumes; however, their findings were not consistent (i.e., poorer outcomes in the older adults with remote mTBIs in one study and no differences between the remote mTBI group and controls in the other study). For the remaining studies, the control groups were either another clinical population (37, 61) or age matched adults without a TBI history (35, 37, 49). No discernable trends were identified across the studies that compared adults with mTBI to other clinical populations. For the studies that compared adults with mTBI to control participants without a mTBI history, findings were also generally disparate. Two of these studies found no group differences (37, 49) while the other found alterations in cerebral blood flow, atrophy in subcortical structures, and other indicators of white matter disruption in several areas in the mTBI group (35). While the diversity of the designs used for examining the long-term neuroimaging findings associated with mTBI in the general population was a strength, the lack of replication of these studies, their correlational design, and inconsistency in findings were important factors that limited their generalizability.

### Repetitive Head Injuries

Some of the studies included in this review focused on examining the potential long-term effects of RHIs on the brain (34, 55, 65–73). One major challenge of this line of research is the variability in defining and capturing RHIs. All studies relied on self-reported proxies of RHIs, including self-reported estimations of years of sport play, estimates of ball-headings, and/or estimates of the number of times players recalled being taken out of play following head impact. None of the included studies used objective measures to support RHI exposure estimates (e.g., accelerometer data or systematic video review). However, one study attempted to quantify RHI exposure by collecting self-reported data about ball-heading exposure during a typical practice and competitive game and then extrapolated this data across a one-year timespan. They reported that history of a previous concussion did not explain differences between groups (i.e., high-, medium-, or low-heading exposure) in their DTI analyses, but estimated RHI exposure did (71).

Importantly, some studies examined adults who had been exposed to both RHIs and mTBI, making it difficult to disentangle the unique outcomes associated with each injury type (67, 69). For other studies, it was unclear whether data about mTBI exposure was collected or accounted for because only the exposure to contact sports, which was used as a proxy for RHI, was discussed (34, 65). One way to avoid this problem would be to exclude those who have reported having a previous mTBI but include those who have experienced repetitive impacts to the head (e.g., heading the ball in soccer). In doing this, one study found that retired professional athletes had greater levels of metabolites (e.g., choline and myo-inositol) many years after their potential RHI exposure compared to controls (73). A similarly designed study (70), found greater cortical thinning (i.e., right inferolateral-parietal, temporal, and occipital cortex) with increasing age in soccer players than controls. However, given the lack of studies that fit these criteria, care must be taken in interpreting these findings. Inferences on a larger scale cannot be made without further research in this area.

### Early vs. Late Life Exposure to Head Injury

Studies that have examined age at first exposure to head injury have reported conflicting findings. Findings from several retrospective studies in this review suggest that sustaining injuries earlier in life (i.e., early adulthood) may be associated with the presence of neuroimaging findings (e.g., smaller regional volumes or white matter disruption) in the long-term compared to experincing a head injury at an older age (44, 49, 57, 65). With more evidence to support findings, this would have important implications for young adults who are at increased risk for sustaining a mTBI (e.g., athletes) or for older adults who were exposed to mTBIs in early adulthood. Importantly, one study identified in this review purported that neurological seqaelae may persist for several years post-injury before resolving (46). With the current methodological limitations to consider at present, more longitudinal research and a greater number of high-quality studies devoted to this topic will be needed to draw conclusions about this matter. Of note, this question was addressed in the general population (49, 57) and in athletes (44, 65) but not in veteran samples.

### Limitations

This systematic review is one of the first to broadly examine the long-term effects of RHIs and mTBIs in several population groups. Given the heterogeneity of the studies included in this review, there are important limitations to consider. Firstly, there were generally small sample sizes (see Figure 2) and a lack of inclusion of other variables (e.g., genetics, substance use, mental health status, medical or psychiatric diagnoses, diet, exercise, sleep habits) that may influence the relationship between RHI or MTBI exposure and neuroimaging findings in the long-term (76). The majority of studies employed a retrospective, cross-sectional design, with some variability in the average time since last injury/exposure to RHIs (see Figure 2). In addition, generalizability of findings was limited by the broad range of imaging data acquisition, preprocessing, and analysis methods used, the lack of replication studies, and the diverse control groups used. Furthermore, there was considerable heterogeneity in the definitions and identification of mTBI and RHIs across studies. Some studies opted for more rigorous methods than others and this should be considered when interpreting findings.

Another limitation to this review was the selection bias for studies that were published in English. In addition, only one study (69) reported entirely on and published null findings (though some reported partial null findings). Considering that studies with null findings are less likely to be published and would therefore be excluded from this review, there is a potential risk for publication bias. This review also included all relevant studies and did not discriminate based on the quality of evidence available. Therefore, it is possible that findings may have been overestimated due to the inclusion of studies with lower evidence. Finally, without post-mortem studies in this review, inferences about susceptibility to neurodegenerative diseases were not possible to address as most studies focussed on risk factors that may be associated with early neuropathology instead.

## Conclusion and Future Directions

Overall, there appears to be some evidence to suggest an association between exposure to mTBI and neuroimaging findings in older adulthood. Though the neuroimaging studies captured in this review were mostly cross-sectional in design, there was some evidence for differences in white matter microstructure (i.e., mainly in the corpus callosum and superior longitudinal fasciculus, though discrepancies were observed) (32, 35, 45, 49, 51, 58, 61, 67, 71), smaller brain structures (34, 35, 37, 45, 53, 55–57, 59, 65, 74), neurochemical differences (62, 66, 73), functional brain differences (32, 33, 45, 47, 57, 72), altered cerebral blood flow (51, 69), and greater proteinopathy (36, 37, 50) in adults who have a history of RHIs and/or one or more mTBIs beyond at least one decade. However, there are a number of limitations within the current literature (see section Limitations). Future research should be devoted to replicating and expanding the present research through addressing some of the methodological concerns highlighted in this systematic review. With the publication of more higher-powered longitudinal studies, it may be possible to better detect neural structures vulnerable to this injury, if any, over time. Based on this review, it is also evident that greater consistency and clarity is needed in the definition and identification of injuries retrospectively. A specific focus on improving our understanding of these associations in the general population and with veterans/service members is also necessary.

Furthermore, more recent studies often employed newer analysis techniques (e.g., region by region volumetric comparisons to more data-driven approaches to analyses) (37, 49, 56), used newer technology (e.g., 0.5T MRI in 1990 versus 3T in 2020) (34, 35), or modalities (e.g., OCT) (52, 63), which further complicates synthesis across studies. Furthermore, future studies should examine the amount of variance accounted for by mTBI/RHIs, beyond that of other factors that may contribute to positive neuroimaging findings or possible accelerated brain aging (e.g., genetics, lifestyle differences, illnesses, etc.). Finally, well-designed longitudinal studies are needed to understand the potential contribution of mTBI and RHIs to the aging process.

## Data Availability Statement

The original contributions presented in the study are included in the article/Supplementary Material, further inquiries can be directed to the corresponding author/s.

## Author Contributions

HE and MW conceived of the presented idea. HE formulated the search strategy, searched the 4 databases, extracted the data, and wrote the manuscript. HE and AR screened all identified titles and abstracts as well as the remaining full texts and assessed the level of evidence and risk of bias for all studies. HE, AR, and MW edited the manuscript and MW supervised the findings of this work. All authors provided critical feedback and helped shape the manuscript.

## Conflict of Interest

The authors declare that the research was conducted in the absence of any commercial or financial relationships that could be construed as a potential conflict of interest.

## Publisher's Note

All claims expressed in this article are solely those of the authors and do not necessarily represent those of their affiliated organizations, or those of the publisher, the editors and the reviewers. Any product that may be evaluated in this article, or claim that may be made by its manufacturer, is not guaranteed or endorsed by the publisher.
