# Supplementary material for: Systematic Review of the Long-Term Neuroimaging Correlates of Mild Traumatic Brain Injury and Repetitive Head Injuries
Source: Front Neurol. 2021 Sep 30;12:726425. doi: 10.3389/fneur.2021.726425 (PMC8514830; doi:10.3389/fneur.2021.726425)
Supplement: Supplementary file 1 [file Data_Sheet_1.docx]

**Systematic review of the long-term neuroimaging correlates of mild traumatic brain injury and repetitive head injuries**

**Online Supplementary Material: Tables 1-3**

Table of Contents

[Supplementary Table 1. MEDLINE search strategy 1](#_Toc66039831)

[Supplementary Table 2. Summary of Studies 2](#_Toc66039832)

[Supplementary Table 2. Downs and Black checklist for the assessment of the methodological quality of both randomized and non-randomized studies. 26](#_Toc66039833)

[Appendix: Downs and Black checklist for the assessment of the methodological quality of both randomized and non-randomized studies. 28](#_Toc66039834)

[References 29](#_Toc66039835)

**Supplementary Table 1. MEDLINE search strategy.**

| **Concussion Terms** | **Neuroimaging Terms** | **Long Term Outcome Terms** |
| --- | --- | --- |
| **MeSH** | **MeSH** | **MeSH** |
| Brain Concussion/ or Brain Damage, Chronic/ or Brain Injuries/ or Brain Injuries, Diffuse/ or Brain Injuries, Traumatic/ or Brain Injury, Chronic/ or Contrecoup Injury/ or Craniocerebral Trauma/ or Diffuse Axonal Injury/ or Head Injuries, Closed/ | Biomarkers/ or Brain Mapping/ or Cell Tracking/ or Cerebral Angiography/ or Cerebral Ventriculography/ or Computed Tomography/ or Computed Tomography Angiography/ or Connectome/ or Diffusion Magnetic Resonance Imaging/ or Diffusion Tensor Imaging/ or Echoencephalography/ or Echo-Planar Imaging/ or Fluorine-19 Magnetic Resonance Imaging/ or Functional Neuroimaging/ or Imaging, Three-Dimensional/ or Magnetic Resonance Imaging/ or Magnetic Resonance Angiography/ or Molecular Imaging/ or Multimodal Imaging/ or Neuroimaging/ or Neuroradiography/ or Positron-Emission Tomography/ or Positron Emission Tomography Computed Single Photon Emission Computed Tomography Perfusion Imaging/ or Radionuclide Imaging/ or Spectroscopy, Near-Infrared/ or Tomography, Emission-Computed/ or Tomography, Emission-Computed, Single-Photon/ or Tomography/ or Tomography, X-Ray/ or Tomography, X-Ray Computed/ or Ultrasonography, Doppler, Transcranial/ | Alzheimer Disease/ or Amyotrophic Lateral Sclerosis/ or Chronic Traumatic Encephalopathy/ or Cognition Disorders/ or Dementia/ or Endocrine System Diseases/ or Frontotemporal Dementia/ or Health Status/ or Hypopituitarism/ or Motor Neuron Disease/ or Neurodegenerative Diseases/ or Parkinson Disease, Secondary/ or Parkinson Disease/ or Septum Pellucidum/ or Tauopathies/ or TDP-43 Proteinopathies/ |
| **Keywords** | **Keywords** | **Keywords** |
| ((post* or cumulat* or mild or multiple or repetitive or subacute or sub-acute) adj3 concuss*).ti,ab,kw,kf.  (((brain or crani* or head* or sport*) adj2 (accident* or damag* or impact* or injur* or trauma*)) or concussi* or postconcussi* or subconcuss* or second impact syndrome or mtbi).ti,ab,kw,kf. | (neural network or white matter or gray matter or grey matter or neural correlates or structural correlates or diffusion correlates or volumetric correlates or functional correlates or subcortical or neural or imaging or MRI or PET or magnetoencephalography or MEG or electroencephalography or EEG or functional magnetic resonance imaging or fMRI or DTI or T2* or diffusion spectrum imaging or DSI or diffusion weighted imaging or DWI or SWI or susceptibility weighted imaging or CT or FLAIR or diffusion* kurtosis imaging or DKI or SPECT or NIRS or fNIRS or functional near-infrared spectroscopy or resting state or functional connectivity or structural connectivity or structural covariance or functional covariance or default mode network).ti,ab,kw,kf. | (acquired seizure disorder or chronic traumatic encephalopath* or cte or neurodegenerative disease* or neurodegenerative disorder* or neuropathol* or neurodegenerat* or dementia* or dementia pugilistica or neurodegenerative dementia* or punch drunk or traumatic encephalopath* or alzheimer* or amyotrophic lateral sclerosis or als or motor neuron disease* or parkinson* or septum pellucidum or (cognit* adj3 impairment) or (cogniti* adj3 deficit*) or white matter tract* or tdp-43 or tauopath* or endocrine dysfunction or hypopituitarism or (long term adj3 impairment) or (long term adj3 sequelae) or sequela*).ti,ab,kw,kf.  (Health adj2 (outcome* or status or effect*)).ti,ab,kw,kf. |

**Supplementary Table 2. Summary of Studies.**

| Lead Author and Study Year | PMID | Study Design | Sample Size | Age: mean (SD) | Sex  M:F | Risk of Bias | Level of Evidence |
| --- | --- | --- | --- | --- | --- | --- | --- |
| (Bryant et al., 2020) | 31587599 | Cross Sectional Study | 64 retired fighters and 442 active fighters | Age of Experimental Group: 47.8 (9.53) for retired fighters and 29.05 (5.44) for active fighters | Sex of Experimental Group: 61:3 for retired fighters and 407:35 for active fighters | 10 | Level 4 |
| **Aim of Study:** To examine the relationship between age of first exposure (AFE) to fighting sports and brain health in later life.  **Key Outcome Measures:** Age at first exposure (self-reported), cognitive testing (CNS vital signs), neurologic examination, psychiatric symptom scales (PHQ-9; BIS-11), and a 3 T magnetic resonance imaging (MRI) scan with structural and functional sequences (main outcomes: volumes of the hippocampus, amygdala, caudate, putamen, and thalamus, and the anterior, central, and posterior corpus callosum).  **Age at Head Injury:** Not Reported, **Years Since Head Injury:** Not reported. The mean years of fighting exposure was 11.52 (5.64) for retired fighters and 5.27 (4.43) for active fighters  **Years of Education:** Median years: 12 for retired fighters and 13 for active fighters; **Race (%):** African American (27%), White (67%), Other (6%); **Population Type:** Athletes  **Diagnosis of Concussion:** Not Reported. This study used years of fighting as proxy for exposure to repetitive head injury (RHI).  **Key Findings:** Brain MRI data showed significant correlations between earlier AFE and smaller bilateral hippocampal and posterior corpus callosum volumes for both retired and active fighters. There was no correlation between AFE and left amygdala volume for retired fighters. However, active fighters showed a correlation between earlier AFE and smaller left amygdala volume. Conversely, there was a correlation between earlier AFE and smaller right amygdala volume for retired fighters, while active fighters showed no correlation. There were no significant correlations between AFE and thalamus, caudate, or putamen volume on either side in either cohort. Likewise, there was no correlation between anterior or central corpus callosum volume and AFE in either cohort. | | | | | | | |
| Lead Author and Study Year | PMID | Study Design | Sample Size | Age: mean (SD) | Sex  M:F | Risk of Bias | Level of Evidence |
| (Casson, Viano, Haacke, Kou, & LeStrange, 2014) | 25177413 | Cross Sectional Study | 45 | Age of Experimental Group: 45.6 (8.9) | Sex of Experimental Group: 45:0 | 8 | Level 3 |
| **Aim of Study:** To assess whether objective clinical abnormalities can be observed in 30- to 60- year-old retired National Football League (NFL) players.  **Key Outcome Measures:** MRI (1.5 T MRI; susceptibility weighted imaging [SWI], diffusion tensor imaging [DTI]), neuropsychological (MMSE, BDI, PHQ, ImPACT) and neurological examinations, interviews, blood tests, BMI (mean: 31.4, SD: 4.8), and APOE genotyping.  **Age at Head Injury:** Not Reported, **Years Since Head Injury:** Mean of 6.8 (3.2) years of NFL experience earlier in life (ages not explicitly stated).  **Years of Education:** 15.5 (1.1); **Race (%):** Not Reported; **Population Type:** Athletes  **Diagnosis of Concussion:** Self-reported mTBIs obtained through detailed neurological and concussion history screening. Participants were provided a standard definition of concussion.  **Key Findings:** The retired players had 6.8 ± 3.2 years (maximum, 14 years) of NFL play and reported 6.9 ± 6.2 concussions (maximum, 25) in the NFL. The majority of retired players had normal clinical mental status and central nervous system (CNS) neurological examinations. Four players (9%) had microbleeds in brain parenchyma identified in SWI, and 3 (7%) had a large cavum septum pellucidum with brain atrophy. The number of concussions/ “dings” was associated with abnormal results in SWI and DTI. SWI detected 4 cases with microbleeds. Anatomical MRI: Two cases were found with abnormally enlarged ventricles and thin corpus callosum, suggesting brain atrophy. Neuropsychological testing revealed isolated impairments in 11 players (24%), but none met criteria for dementia. Nine players (20%) endorsed symptoms of moderate or severe depression on the BDI and/or met criteria for depression on PHQ; however, none had dementia, dysarthria, parkinsonism, or cerebellar dysfunction. The number of football-related concussions was associated with isolated abnormalities on the clinical neurological examination, suggesting CNS dysfunction. There was a statistical association between the presence of abnormalities on the clinical CNS examination and the total number of football concussions sustained at all levels of play. | | | | | | | |
| Lead Author and Study Year | PMID | Study Design | Sample Size | Age: mean (SD) | Sex  M:F | Risk of Bias | Level of Evidence |
| (Chong, Peplinski, Ross, & Berisha, 2018) | 30913910 | Cross Sectional Study | 131 total (138 before exclusions due to imaging abnormalities) | Age of Experimental Group: Migrane group: 39.1 (10.7) and PPTH group: 37.7 (10.6)  Age of Control Group: 38.1 (10.2) | Sex of Experimental Group: Migrane group: (15:26) and PPTH group: (32:17)  Sex of Control Group: 22:19 | 9 | Level 4 |
| **Aim of Study:** To determine changes in fibertract profiles that are unique to either migraine or to persistent post-traumatic headache (PPTH), and to better understand whether these changes relate to disease characteristics, by exploring the relationships between headache frequency and alterations in fibertract profiles.  **Key Outcome Measures:** Headache history, the Beck Depression Inventory (BDI), the State Anxiety Inventory, and the Ohio State University TBI Identification questionnaire. DTI and a novel method for detecting subtle changes in fibertract integrity by measuring node-by-node parameters along each tract to compare fibertract profiles between those with migraine and those with PPTH, and compared both cohorts to a group of controls. Node-by-node diffusion parameters of mean diffusivity (MD) and radial diffusivity (RD) were calculated along each tract.  **Age at Head Injury:** Not Reported, **Years Since Head Injury:** 10 (8.1) for the PPTH group  **Years of Education:** Not Reported; **Race:** Not Reported; **Population Type:** General Population  **Diagnosis of Concussion:** Ohio State University TBI Identification questionnaire for determining history of TBI was used.  **Key Findings:** There were significant differences between migraine and persistent post-traumatic headache cohorts for quartile measurements of MD or RD in the bilateral anterior thalamic radiations, cingulum (angular bundles and cingulate gyri), inferior longitudinal fasciculi, and uncinate fasciculi, the left corticospinal tract, and the right superior longitudinal fasciculi-parietal portion. For migraine patients, there was a significant positive correlation between headache frequency and forceps major MD, whereas for persistent post-traumatic headache there was a positive correlation between headache frequency and cingulum angular bundle MD and RD. | | | | | | | |
| Lead Author and Study Year | PMID | Study Design | Sample Size | Age: mean (SD) | Sex  M:F | Risk of Bias | Level of Evidence |
| (Clark et al., 2018) | 29087238 | Cross Sectional Study | 61 | Age of Experimental Group: Professional players: 3/more TBI (n = 15): 57.9 (3.74); 0-1 TBI (n=15): 59.2 (3.55).  Age of Control Group: College players: 3/more TBI (n = 16): 58.2 (3.43); 0-1 TBI (n=15): 58.9 (4.12). | Sex of Experimental Group: College players: 31:0 and professional players: 30:0 | 14 | Level 4 |
| **Aim of Study:** To better understand the relationship between exposure to concussive and subconcussive head impacts, white matter integrity, and functional task-related neural activity in former U.S. football athletes (NFL and college players).  **Key Outcome Measures:** Participants were stratified across three crossed factors: career duration, concussion history, and primary playing position. Fractional anisotropy (FA) and blood oxygen level-dependent (BOLD) percent signal change (PSC) were measured with diffusion-weighted and task-related functional MRI (fMRI), respectively. Analyses of variance of FA and BOLD PSC were used to determine main or interaction effects of the three factors.  **Age at Head Injury:** Not Reported, **Years Since Head Injury:** Estimated (>10) based on years of exposure to football which for the professional players in the 3 or more concussions group (n = 15) was: 17.6 (3.86), and for the 0-1 concussions group (n = 15) was: 17.7 (2.53); college players in the 3 or more concussions group (n = 16): 8.06 (0.68), and the 0-1 concussions group (n = 15): 8.05 (0.60).  **Years of Education:** Professional players: the 3 or more concussions group (n = 15): 16.7 (0.976) and the 0-1 concussions group (n = 15): 16.7 (1.45). For the College players: the 3 or more concussions group (n = 16): 16.6 (1.03) and the 0-1 concussions group (n = 15): 16.4 (0.828); **Race (%):** Not Reported; **Population Type:** Athletes  **Diagnosis of Concussion:** Self-reported while provided a standard definition of concussion (McCrory et al., 2013). Exposure to subconcussive impacts was estimated by football career duration (i.e., retired after college football or coninuied to professional foooball for 5+ years.)  **Key Findings:** A significant interaction between career duration and concussion history was observed; former college players with more than three concussions had lower FA in a broadly distributed area of white matter compared with those with zero to one concussion (t(29) = 2.774; adjusted p = .037), and the opposite was observed for former professional players (t(29) = 3.883; adjusted p = .001). A separate interaction between concussion history and position was observed: Nonspeed players (i.e., offensive or defensive linemen) with more than three concussions had lower FA in frontal white matter compared with those with zero to one concussion (t(25) = 3.861; adjusted p = .002). Analysis of working memory-task BOLD PSC revealed a similar interaction between concussion history and position (all adjusted p < .004). Former football athletes who played a nonspeed position (ie, offensive or defensive linemen) and reported three or more concussions had lower FA (t(25) = 3.861; adjusted p = .002) and lower blood oxygen level-dependent percent signal change during a working memory task (t(25) = 3.537; adjusted p = .004) than did those with zero to one concussion. | | | | | | | |
| Lead Author and Study Year | PMID | Study Design | Sample Size | Age: mean (SD) | Sex  M:F | Risk of Bias | Level of Evidence |
| (De Beaumont et al., 2013) | 23972282 | Cross Sectional Study | 30 | Age of Experimental Group: 60.87 (7.51)  Age of Control Group: 58.13 (5.28) | Sex of Experimental Group: 15:0  Sex of Control Group: 15:0 | 10 | Level 4 |
| **Aim of Study:** To examine how aging combined with a history of remote concussions interact to affect M1 metabolism and function in former university-level athletes. This study also sought to determine whether neurometabolic abnormalities are observed in M1 of former concussed athletes, and if so, whether they relate to possibly reduced M1-dependent motor sequence learning.  **Key Outcome Measures:** They used proton magnetic resonance spectroscopy (3 T MRI) to detect metabolic abnormalities in the primary motor cortex and the serial reaction time task (SRTT) to evaluate motor learning.  **Age at Head Injury:** Not Reported, **Years Since Head Injury:** 37.07 (7.93)  **Years of Education:** Controls: 17.27 (3.45), concussed group: 16.67 (4.07); **Race (%):** Not Reported; **Population Type:** Athletes (former university level hockey and football athletes)  **Diagnosis of Concussion:** Self-reported and collected by a certified neuropsychologist (according to the American Academy of Neurology practice parameters). All classified as mild traumatic brain injury on the Glasgow Coma Scale (scoring between 13 to 15).  **Key Findings:** Among concussed athletes, they found that the number of concussions sustained was negatively correlated with relative M1 glutamate/H_2_O levels (r = -.631; P = .021) as well as sequence-specific motor learning (r = -.597; p = .029). They also found a significant Age by Group interaction for M1 glutamate concentration (F1, 24= 4.616; P = .048; ηp2 = 0.755), indicating that the effect of aging on M1 glutamate levels was significantly exacerbated in former concussed athletes. In contrast, the Age by Group interaction for NAA levels in M1 did not reach significance (F1, 24 = 3.50; P = .074; ηp2 = 0.132). | | | | | | | |
| Lead Author and Study Year | PMID | Study Design | Sample Size | Age: mean (SD) | Sex  M:F | Risk of Bias | Level of Evidence |
| (Ford, Giovanello, & Guskiewicz, 2013) | 23679098 | Cross Sectional Study | 41 | Age of Experimental Group: 62.6 (5) for the high concussion group and 64.1 (6.8) for the low concussion group).  Age of Control Group: 62.2 (6.3) | Sex of Experimental Group: 12:0 in the high concussion group and 15:0 in the low concussion group  Sex of Control Group: 14:0 | 11 | Level 4 |
| **Aim of Study:** To use event-related fMRI to examine long-term neural changes associated with multiple sport-related concussions.  **Key Outcome Measures:** Event-related fMRI was used to examine long-term differences in neural activity during memory tasks in former athletes who sustained multiple sport-related concussions. Cognitive measures included: the telephone interview for cognitive status, MMSE, WAIS, COWA, TMT-B, BNT, WTAR, and GDS.  **Age at Head Injury:** Not Reported, **Years Since Head Injury:** Not Reported (more than 10)  **Years of Education:** Controls: 16.4 (1.1), low-concussion group: 16.4 (1.4), and high-concussion group: 16.2 (1.3); **Race (%):** Not Reported; **Population Type:** Athletes (former professional NFL players with minimum two seasons of play and healthy aged matched controls)  **Diagnosis of Concussion:** Self-reported (method not described)  **Key Findings:** Concussion history was not significantly associated with cognitive performance. In contrast, the two groups of former players demonstrated different neural recruitment patterns during relational memory retrieval. In addition, the number of previous concussions significantly correlated with functional activity in a number of brain regions, including the medial temporal lobe and inferior parietal lobe. | | | | | | | |
| Lead Author and Study Year | PMID | Study Design | Sample Size | Age: mean (SD) | Sex  M:F | Risk of Bias | Level of Evidence |
| (Gardner et al., 2016) | 25970145 | Cross Sectional Study | 34 | Age of Experimental Group: 54.6 (15.8)  Age of Control Group: 54.7 (15.8) | Sex of Experimental Group: 17:0  Sex of Control Group: 17:0 | 13 | Level 4 |
| **Aim of Study:** To characterize neuroimaging features of the septum pellucidum in a cohort of retired American pro-football players presenting with cognitive/behavioral symptoms compared with similar memory clinic patients without a history of TBI.  **Key Outcome Measures:** CSP grade was measured with 3 T structural MRI (CSP rated as 0-absent, 1-equivocal, 2-mild, 3-moderate, 4-severe) and length was measured according to a standard protocol.  **Age at Head Injury:** Not Reported, **Years Since Head Injury:** Not Reported. Average number of years since retired from pro-football: 24.5 (15.5). Total lifetime exposure to football since childhood (in years): 17.3 (4.5).  **Years of Education:** Controls: 17.3 (5.2) and players: 17.7 (3.3); **Race (%):** Not Reported; **Population Type:** Athletes (retired pro-football players with at least one season of play)  **Diagnosis of Concussion:** Self-reported (method not described)  **Key Findings:** They retrospectively assessed retired American pro-football players presenting to a memory clinic with cognitive/behavioral symptoms. Each player was matched to a memory clinic control patient with no history of TBI. Sixteen of 17 (94%) players had a CSP graded >2 compared with 3 of 17 (18%) controls. CSP was significantly higher grade (p < 0.001) and longer in players than controls (mean length-standard deviation: 10.6 mm-5.4 vs. 1.1 mm-1.3, p < 0.001). Results were similar for CSP length, total septal cyst length, and their ratios, suggesting that results were not confounded by larger head sizes among players. CSP >2 distinguished players from controls with 94% sensitivity (95% confidence interval [CI] 71-100%) and 82% specificity (95% CI 57-96%). CSP length of 5 mm distinguished players from controls with 82% sensitivity (95% CI 57-96%) and 100% specificity (95% CI 81-100%). | | | | | | | |
| Lead Author and Study Year | PMID | Study Design | Sample Size | Age: mean (SD) | Sex  M:F | Risk of Bias | Level of Evidence |
| (Gilmore et al., 2020) | 33351088 | Longitudinal Cohort Study | 139 | Age of Experimental Group: 50 (10.3)  Age of Control Group: 49.9 (11.9) | Sex of Experimental Group: 62:7  Sex of Control Group: 55:15 | 10 | Level 4 |
| **Aim of Study:** To identify evidence of neurodegeneration through longitudinal evaluation of structural and functional changes in the visual nervous system and CNS in veterans with a history of mild TBI.  **Key Outcome Measures:** Change over time of retinal nerve fibre layer (RNFL) thickness using optical coherence tomography. Cognitive tests: CogState battery and Groton Maze Learning Test (GMLT).  **Age at Head Injury:** Not Reported, **Years Since Head Injury:** median: 17 years  **Years of Education:** Not Reported in number of years; **Race (%):** Not Reported; **Population Type:** Veterans  **Diagnosis of Concussion:** mTBI was diagnosed according to the Mayo TBI Severity Classification System.  **Key Findings:** Veterans with mTBI showed significantly greater RNFL thinning compared with controls. RNFL tissue loss was significantly correlated with both worsening performance on the GMLT over time (Spearman ρ = −0.20; p =.03) and mTBI severity (Spearman ρ = −0.25; p = .006). The more severe the mTBI (larger Minnesota Blast Exposure Screening Tool severity score), the faster the reduction in RNFL thickness (i.e., the more negative the slope) across time. | | | | | | | |
| Lead Author and Study Year | PMID | Study Design | Sample Size | Age: mean (SD) | Sex  M:F | Risk of Bias | Level of Evidence |
| (Goswami et al., 2016) | 25721800 | Case Series | 36 | Age of Experimental Group: 50 (12)  Age of Control Group: 46 (10) | Sex of Experimental Group: 19:0  Sex of Control Group: 17:0 | 9 | Level 4 |
| **Aim of Study:** The first aim was to determine the relationship between response inhibition and related psychological factors to the structural and functional properties of the uncinate fasciculus (UF) and frontotemporal gray matter in retired professional athletes (CFL players) with a chronic history of multiple concussions. Machine learning was also used to test the predictive power of diffusion imaging metrics within the UF to discriminate these concussed athletes from controls.  **Key Outcome Measures:** Used MRI (DWI and rs-fMRI) to examine the UF and connected gray matter as it relates to impulsivity (SART: go/no go task) and aggression (the PAI). Examined cortical thickness, diffusion, probabilistic tractography (including registration of tractography maps for machine learning analysis), resting state fMRI, and machine learning for DTI metrics.  **Age at Head Injury:** Not Reported. Almost half reported their first concussion was in high school, **Years Since Head Injury:** Not Reported  **Years of Education:** Experimental group: 17 (1.8), controls: 16 (1.9); **Race (%):** Not Reported; **Population Type:** Athletes  **Diagnosis of Concussion:** Self-reported. Concussion was operationally defined in accordance with the guidelines agreed upon by the International Consensus statements (McCrory et al., 2013; Tator, 2013).  **Key Findings:** Behaviourally, athletes had faster reaction times and an increased error rate on a go/no-go task, and increased aggression and mania on the PAI compared to controls. MRI revealed that the athletes had (1) cortical thinning of the anterior temporal lobe (ATL), (2) negative correlations of orbital frontal cortex (OFC) thickness with aggression and task errors, indicative of impulsivity, (3) negative correlations of UF axial diffusivity (AD) with error rates and aggression, and (4) elevated resting-state functional connectivity between the ATL and OFC. Using machine learning, they found that UF diffusion imaging differentiates athletes from healthy controls with significant classifiers based on UF MD and RD showing 79-84 % sensitivity and specificity, and 0.8 areas under the ROC curves. The spatial pattern of classifier weights revealed hot spots at the orbitofrontal and temporal ends of the UF. | | | | | | | |
| Lead Author and Study Year | PMID | Study Design | Sample Size | Age: mean (SD) | Sex  M:F | Risk of Bias | Level of Evidence |
| (Haglund & Bergstrand, 1990) | 2281746 | Cross Sectional Study | 100 | Age of Experimental Group: HM-boxers (mean: 32.6 years), LM-boxers (mean: 33.6 years)  Age of Control Group: SP mean age: 33, TF mean age: 33.4 | Sex of Experimental Group: HM-group: 25:0, LM-group 25:0  Sex of Control Group: SP group: 25:0, TF group 25:0 | 8 | Level 4 |
| **Aim of Study:** To find out if morphological changes could be found in former amateur (low match and high match) boxers using CT and MRI.  **Key Outcome Measures:** CT and MRI for evaluation of a cavum septum pellucidum (CSP).  **Age at Head Injury:** Not Reported, **Years Since Head Injury:** Average length of career for HM group: 8.3 years and 3.4 years for LM group.  **Years of Education:** Not Reported; **Race (%):** Not Reported; **Population Type:** Athletes (former amateur boxers, soccer and track and field players as controls).  **Diagnosis of Concussion:** Not described. Years of fighting experience was used as a proxy for repetitive brain injury.  **Key Findings:** No significant differences in the width of the ventricular system, anterior horn index, width of cortical sulci, signs of vermian atrophy, or the occurrence of a CSP were found between boxers and controls. A higher incidence of CSP was found in track and field athletes than in the other two groups who sustained repeated head trauma (i.e. boxers and soccer players). The authors state that a CSP is probably an anatomical normal variation and that it can not be concluded from this study that it is a sign of earlier head trauma. Although a correlation existed between the occurrence of a CSP and the number of fights, fights lost, KO/RSC(H) and the length of boxing career, it should be noted that there were only two boxers who had a CSP. | | | | | | | |
| Lead Author and Study Year | PMID | Study Design | Sample Size | Age: mean (SD) | Sex  M:F | Risk of Bias | Level of Evidence |
| (Hampshire, MacDonald, & Owen, 2013) | 24135857 | Cross Sectional Study | 33 | Age of Experimental Group: 54  Age of Control Group: 53 | Sex of Experimental Group: 13:0  Sex of Control Group: 20:0 | 6 | Level 4 |
| **Aim of Study:** To assess the performances and functional brain activations of a group of retired NFL players whilst undertaking the One Touch Spatial Planning task, an fMRI-optimised variant of the classical Tower of London paradigm that is widely used to assess executive cognition.  **Key Outcome Measures:** Evaluated the performances and brain activation patterns of retired NFL players (NFL alumni) relative to controls using an fMRI-optimised neuropsychological test of executive function.  **Age at Head Injury:** Not Reported, **Years Since Head Injury:** Not Reported.  **Years of Education:** Not Reported; **Race (%):** Not Reported; **Population Type:** Athletes (retired NFL and controls)  **Diagnosis of Concussion:** Self-reported number of times that they had been taken out of play due to head impact.  **Key Findings:** Retired NFL players showed modest performance deficits on the executive task but demosntrated pronounced hyperactivation and hypoconnectivity of the dorsolateral frontal and frontopolar cortices. The NFL alumni exhibited pronounced hyperactivation within the same DLPFC regions that were engaged by controls during planning and retrieval. This hyperactivation was evident at all levels of planning complexity and for all levels of working-memory load, including those for which there was no significant difference in performance. Moreover, frontopolar regions were selectively recruited in the NFL alumni during more difficult trials whereas in controls no such activation was evident. Abnormal frontal-lobe function was correlated with the number of times that NFL alumni reported having been removed from play after head injury and was evident in individual players. | | | | | | | |
| Lead Author and Study Year | PMID | Study Design | Sample Size | Age: mean (SD) | Sex  M:F | Risk of Bias | Level of Evidence |
| (Hart et al., 2013) | 23303193 | Cross Sectional Study | 48 | Age of Experimental Group: unimpaired retired NFL players mean age: 55.4, cognitively impaired retired NFL players 66.6  Age of Control Group: 60.1 | Sex of Experimental Group: unimpaired retired NFL players: 12:0 and cognitively impaired retired NFL players: 10:0  Sex of Control Group: 26:0 | 9 | Level 4 |
| **Aim of Study:** To assess cognitive impairment and depression in aging former NFL players and to identify neuroimaging correlates of these dysfunctions.  **Key Outcome Measures:** Neuropsychological measures, clinical diagnoses of depression, neuroimaging (3 T MRI) measures of white matter pathology (DTI), and a measure of cerebral blood flow (pseudocontinuous arterial spin labelling and Hemosiderin Scan).  **Age at Head Injury:** Not Reported, **Years Since Head Injury:** Estimated (>10) based on years of NFL exposure several decades ago, which was an average of 9.7 years.  **Years of Education:** Unimpaired retired NFL players mean years: 16.6, cognitively impaired retired NFL players: 16.1, controls: 16.2; **Race (%):** 23 of the retired NFL players were white and 11 were African American. Control group: 24 were white, 2 were African American.; **Population Type:** Athletes (former NFL and healthy mattched controls)  **Diagnosis of Concussion:** Concussion history was obtained retrospectively from participants and informants and classified using the 1997 American Academy of Neurology practice parameter guidelines.  **Key Findings:** Of the 34 former NFL players, 20 were without cognitive impairments. Four were diagnosed as having a fixed cognitive deficit; eight, mild cognitive impairment; two, dementia; and eight, depression. Of the subgroup in whom neuroimaging data were acquired, cognitively impaired participants showed the greatest deficits on tests of naming, word finding, and visual/verbal episodic memory. Significant differences were found in white matter abnormalities in cognitively impaired and depressed retired players compared with their respective controls (they found widely distributed reductions of FA in frontal and parietal regions bilaterally as well as along the corpus callosum and in the left temporal lobe). Regional blood flow differences in the cognitively impaired group (impaired players had decreased blood flow to the left temporal pole and increased blood flow to the inferior parietal lobule and superior temporal gyrus) corresponded to regions associated with impaired neurocognitive performance (problems with memory, naming, and word finding). | | | | | | | |
| Lead Author and Study Year | PMID | Study Design | Sample Size | Age: mean (SD) | Sex  M:F | Risk of Bias | Level of Evidence |
| (June et al., 2020) | 32702483 | Longitudinal Cohort Study | 153 | Age of Experimental Group: 65.14 (11.23)  Age of Control Group: 65.2 (11.25) | Sex of Experimental Group: 29:12  Sex of Control Group: 58 (56.9) | 11 | Level 3 |
| **Aim of Study:** To examine baseline and longitudinal brain alterations more than 20 years post-concussive event and explore the long-term impact of concussion on subsequent neurodegeneration, in cognitively normal older adults from the Baltimore Longitudinal Study of Aging (BLSA).  **Key Outcome Measures:** Participants underwent serial structural MRI and DTI to measure brain structure, as well as 15 O-water PET to measure brain function. A battery of neuropsychological tests was also administered.  **Age at Head Injury:** 33.05 (22.25), **Years Since Head Injury:** 23 (18.64)  **Years of Education:** Not Reported; **Race (%):** Not Reported by race; **Population Type:** General Population  **Diagnosis of Concussion:** Self-reported in patient's medical history. If loss of consciousness was reported, only those with reports of < 30 min unconsciousness were included in the analyses.  **Key Findings:** Compared to those without concussion, participants with a prior concussion had greater brain atrophy in temporal lobe white matter and hippocampus at first imaging visit, which remained stable throughout the follow-up visits. Those with prior concussion also showed differences in white matter microstructure using DTI, including increased RD and AD in the fornix/stria terminalis, anterior corona radiata, and superior longitudinal fasciculus at first imaging visit. In 15O-water PET, higher resting cerebral blood flow was seen at first imaging visit in orbitofrontal and lateral temporal regions, and both increases and decreases were seen in prefrontal, cingulate, insular, hippocampal, and ventral temporal regions with longitudinal follow-up. There were no significant differences in neuropsychological performance between groups. | | | | | | | |
| Lead Author and Study Year | PMID | Study Design | Sample Size | Age: mean (SD) | Sex  M:F | Risk of Bias | Level of Evidence |
| (Kelman, Hodge, Stanwell, Mustafic, & Fraser, 2020) | 31691473 | Cross Sectional Study | 13 | Age of Experimental Group: mean age of 60 | Sex of Experimental Group: 13:0 | 9 | Level 4 |
| **Aim of Study:** To evaluate retinal nerve fibre layer (RNFL) thickness in professional rugby league players, with RNFL thinning serving as a potential proxy for wider white matter degeneration.  **Key Outcome Measures:** RNFL thickness of each eye (using binocular RNFL thickness as measured by spectral domain optical coherence tomography [OCT]) were compared with a normative database. Other measures included the National Eye Institute Visual Function Questionnaire (NEI-VFQ-25). The Quick Mild Cognitive Impairment (“QMCI”) screen was used to assess cognitive domains. The Depression Anxiety Stress Scale (“DASS-21”) survey was used to assess mood. Alcohol consumption and drinking behaviours were assessed through clinical interview using the Alcohol Use Disorders Identification Test (“AUDIT”).  **Age at Head Injury:** The average age of first concussion was 18, with the earliest reported concussion being at age 11, **Years Since Head Injury:** All participants had competed in Rugby League at a professional club level for 18 years on average. Mean age of introduction to the sport was nine. Average number of years (at testing) since last professional play was 28. Average training per week was four days whilst active, with ten participants reporting weekly games and three participants reporting fortnightly games.  **Years of Education:** Not Reported; **Race (%):** All caucasian; **Population Type:** Athletes  **Diagnosis of Concussion:** Self-reported concussion exposure was evaluated in line with guidelines from the third International Conference on Concussion in Sport (McCrory et al., 2009). A detailed medical and sporting history was taken using a standardized questionnaire.  **Key Findings:** Participants reported sustaining 15 sports-related concussions throughout their career. The RNFL in participants was four micrometres thinner than that of matched normative data. Cohort average RNFL thickness was reduced in 12 out of 14 optical coherence testing parameters. These findings were statistically significant in the left inferonasal [p = .013] and left nasal [p = .006] sectors. | | | | | | | |
| Lead Author and Study Year | PMID | Study Design | Sample Size | Age: mean (SD) | Sex  M:F | Risk of Bias | Level of Evidence |
| (Koerte et al., 2015) | 25843317 | Cross Sectional Study | 25 | Age of Experimental Group: 52 (6.8)  Age of Control Group: 46.9 (7.9) | Sex of Experimental Group: 11:0  Sex of Control Group: 14:0 | 10 | Level 4 |
| **Aim of Study:** To evaluate neurochemistry by using MRS in former professional soccer players without a known history of concussion, but with a history of extensive heading and associated repetitive subconcussive head injury (RSHI), compared with former professional, age-matched, non-contact sport athletes. In addition, the association between brain chemical concentrations, neurocognitive performance, and estimated number of headers was assessed.  **Key Outcome Measures:** 3T magnetic resonance spectroscopy (MRS) neurochemicals quantified: NAA, creatine (Cr), Cho, Glu, GSH, and mI. In addition, lipid and macromolecule resonances were also characterized at 0.9, 1.3, and 2.0 ppm. 11 study participants underwent a brief neurocognitive and balance examination by an examiner who was blinded to the athlete’s sport. Other tests: Trailmaking Test (TMT) parts A and B, Rey-Osterrieth Complex Figure (ROCF) test, and Balance Error Scoring System (BESS).  **Age at Head Injury:** Mean age when soccer training started was 11.5 (4.3), **Years Since Head Injury:** Not Reported  **Years of Education:** Not Reported; **Race (%):** Not Reported; **Population Type:** Athletes  **Diagnosis of Concussion:** Lifetime exposure to soccer was used as an indicator of RSHI exposure (only used players without a history of suspected/diagnosed concussion).  **Key Findings:** In the soccer players a significant increase was observed in both choline (Cho), a membrane marker, and myo-inositol (ml), a marker of glial activation, compared with control athletes. Additionally, ml and glutathione (GSH) were significantly correlated with lifetime estimate of RSHI within the soccer group. It is noteworthy that GSH/Cr levels were significantly correlated with exposure to RSHI. | | | | | | | |
| Lead Author and Study Year | PMID | Study Design | Sample Size | Age: mean (SD) | Sex  M:F | Risk of Bias | Level of Evidence |
| (Koerte, Hufschmidt, et al., 2016) | 26414478 | Cross Sectional Study | 86 | Age of Experimental Group: 54.53 (8.03)  Age of Control Group: former professional noncontact sport athletes: 57.14 (7.62) | Sex of Experimental Group: 72:0  Sex of Control Group: former professional noncontact sport athletes: 14:0 | 10 | Level 4 |
| **Aim of Study:** The aims of this study were (1) to characterize neuroimaging features of CSP in former NFL players who presented with cognitive, mood, and behavioral symptoms compared with asymptomatic noncontact sports athletes, and (2) to evaluate the association between CSP and cognitive and behavioral functioning in former NFL players who present with cognitive, mood, and behavioral symptoms.  **Key Outcome Measures:** High-resolution structural 3T MRI for characterization of CSP. All subjects were administered: Neuropsychological Assessment Battery (NAB) List Learning; Map Reading; Naming; ROCF; TMT, Parts A and B; WAIS-R Digit Symbol; WRAT-4 Reading Test; and the Wisconsin Card Sort Test (WCST). The Hamilton Depression Rating Scale (HAM-D), Brown-Goodwin Lifetime History of Aggression (LHA), Barratt Impulsivity Scale (BIS), and Modified Scale for Suicidal Ideation (MSSI) were assessed as part of the psychiatric interview, while the Behavior Rating Inventory of Executive Function-Adult Version (BRIEF-A), BDI, Beck HopelessnessInventory (BHI), and Buss-Durkee Hostility Inventory (BDHI) were self-completed by each subject.  **Age at Head Injury:** Not Reported, **Years Since Head Injury:** Estimated (>10) based on years of football exposure from several decades ago. Total years of football = 18.11 (3.49) and total years in the NFL = 7.8 (2.67). Also reported the number of concussions: 446.9 (1077.4)  **Years of Education:** NFL players:16.39 (0.87), controls: 17.64 (2.1); **Race (%):** Not Reported; **Population Type:** Athletes  **Diagnosis of Concussion:** Self-reported after receiving a definition of concussion using methods described by Robbins et al (2014).  **Key Findings:** A higher rate of CSP, a greater length of CSP, as well as a greater ratio of CSP length to septum length was found in symptomatic former professional football players compared with athlete controls. In addition, a greater length of CSP was associated with decreased performance on a list learning task (NAB List A Immediate Recall, p = 0.04) and decreased test scores on a measure of estimate verbal intelligence (WRAT-4, p = 0.02). | | | | | | | |
| Lead Author and Study Year | PMID | Study Design | Sample Size | Age: mean (SD) | Sex  M:F | Risk of Bias | Level of Evidence |
| (Koerte, Mayinger, et al., 2016) | 26286826 | Cross Sectional Study | 30 | Age of Experimental Group: 49.3 (5.1)  Age of Control Group: former professional non-contact sport athletes: 49.6 (6.4) | Sex of Experimental Group: 15:0  Sex of Control Group: former professional non-contact sport athletes: 15:0 | 10 | Level 4 |
| **Aim of Study:** To evaluate the association between cortical thickness and estimated exposure to repetitive subconcussive head impact, as well as to cognitive performance in former professional soccer players compared to age-matched non-contact sport athletes.  **Key Outcome Measures:** Cortical thickness analyses from images obtained from a 3 T MRI scanner (using voxel-based statistics). All study participants were evaluated on cognitive, behavioral, and motor functioning by an examiner who was blinded to their sport. Tests included: Trail making Test (TMT) parts A and B, Rey Osterrieth Complex Figure (ROCF), Barrett Impulsivity Score (BIS), and the BESS. TMT parts A and B were performed to quantify the athlete’s psychomotor speed, visual search, and mental flexibility.  **Age at Head Injury:** Not Reported: soccer exposure since childhood was reported in all players, **Years Since Head Injury:** Not Reported  **Years of Education:** Not Reported; **Race (%):** Not Reported; **Population Type:** Athletes  **Diagnosis of Concussion:** Years of playing soccer and estimated ball heading exposure was used as a proxy for repetitive subconcussive head injury.  **Key Findings:** Soccer players demonstrated greater cortical thinning with increasing age compared to controls in the right inferolateral-parietal, temporal, and occipital cortex. Cortical thinning was associated with lower cognitive performance as well as with estimated exposure to RSHI. Neurocognitive evaluation revealed decreased memory performance in the soccer players compared to controls. | | | | | | | |
| Lead Author and Study Year | PMID | Study Design | Sample Size | Age: mean (SD) | Sex  M:F | Risk of Bias | Level of Evidence |
| (Kuhn, Zuckerman, Solomon, Casson, & Viano, 2017) | 30043690 | Cross Sectional Study | 45 | Age of Experimental Group: 46.7 (9.1) | Sex of Experimental Group: 45:0 | 10 | Level 4 |
| **Aim of Study:** To assess the relationships among 3 neuroimaging findings (cavum septum pellucidum, FA global mean, and microhemorrhages) and neuropsychological test performance and symptom endorsement in a relatively large sample of retired NFL athletes.  **Key Outcome Measures:** MRI was performed (anatomical to check for CSP, DTI, and SWI) in 45 retired NFL players. Three neuroanatomical parameters were assessed: (1) the absence or presence of small or large cavum septum pellucidum, (2) a global mean score of FA, and (3) the presence or absence of microhemorrhages. The subjects underwent a battery of 9 neuropsychological tests (i.e., TOMM; BVMT-R; CVLT-II; TMT A and B, WAIS-3 Digit Symbol and Letter Number Sequencing ; COWAT; WTAR, a computerized neurocognitive test (ImPACT), and multiple symptom and depression scales.  **Age at Head Injury:** Not Reported, **Years Since Head Injury:** Not Reported: data not available (but players began their career in their 20's).  **Years of Education:** Not Reported; **Race (%):** Not Reported; **Population Type:** Athletes  **Diagnosis of Concussion:** Self-reported: number of concussions and number of “dings” (defined as “a momentary abnormal sensation in the head occurring immediately upon head impact, with complete resolution within a few seconds and no residual effects”).  **Key Findings:** The 45 subjects reported a mean 6.9 (±6.2) concussions and 13.0 (±7.9) “dings” in the NFL. Ten (22%) did not have a cavum septum pellucidum, while 32 (71%) had a small and 3 (7%) had a large one. Four (9%) had microhemorrhages. Global FA mean was 0.459 (±0.035). The majority (50.8%) of correlations among the neuroimaging parameters and neurocognitive/symptom scores fell below the threshold of “small” effect size (r < 0.10). The remaining (49.2%) correlations were between “small” and “medium” effect sizes (0.1 < r < 0.3). However, all correlations were statistically nonsignificant. The MRI measures of cavum septum pellucidum, FA mean, and microhemorrhaging accounted for between 0.0009% and 6.53% of the variance observed. | | | | | | | |
| Lead Author and Study Year | PMID | Study Design | Sample Size | Age: mean (SD) | Sex  M:F | Risk of Bias | Level of Evidence |
| (Lepage et al., 2019) | 29779184 | Cross Sectional Study | 108 | Age of Experimental Group: 54.86 (7.9)  Age of Control Group: 57.3 (7) | Sex of Experimental Group: 86:0  Sex of Control Group: 22:0 | 10 | Level 4 |
| **Aim of Study:** To compare the volumes of the amygdala, hippocampus, and cingulate gyrus in symptomatic former NFL players, relative to asymptomatic controls without a history of RHI or brain trauma.  **Key Outcome Measures:** Neuroimaging data (volumetric) was acquired on a 3-Tesla MRI Scanner. Regions of interest included the cingulate gyrus, hippocampus, and amygdala. As part of DETECT, participants completed: TMT A and B; Digit Span and Digit Symbol Coding (from the WAIS-R); Wisconsin Card Sorting Test (WCST); Controlled Oral Word Association Test (COWAT); Animal Fluency; Color-Word Interference subtest (DKEFS); ROCF; and Story Learning, List Learning, Naming, and Map Reading, from the NAB. Mood/behavior measures: Apathy Evaluation Scale (AES), BIS-11, BDI-II, Beck Hopelessness Scale (BHS), BRIEF-A, LHA, Center for Epidemiologic Studies - Depression Scale (CES-D), Buss-Durkee Inventory, HAM-D, and the MSSI.  **Age at Head Injury:** Not Reported, **Years Since Head Injury:** Not Reported. Estimated (>10) based on NFL exposure of at least 12 years earlier in life.  **Years of Education:** NFL players: 16.4 (0.96) and for the controls: 17.4 (2.2); **Race (%):** Not Reported; **Population Type:** Athletes  **Diagnosis of Concussion:** Based on self-report after being provided a definition of concussion (Robbins et al., 2014)  **Key Findings:** They compared select limbic brain regional volumes (the amygdala, hippocampus, and cingulate gyrus) between symptomatic former NFL players (n = 86) and controls (n = 22). Compared to controls (with no TBI or contact-sport exposure), former NFL players exhibited reduced volumes bilaterally of the amygdala, hippocampus, and cingulate gyrus. Within the NFL group (n = 75), reduced bilateral cingulate gyrus volume was associated with worse attention and psychomotor speed (r = 0.4 (right), r = 0.42 (left); both p < 0.001), while decreased right hippocampal volume was associated with worse visual memory (r = 0.25, p = 0.027). | | | | | | | |
| Lead Author and Study Year | PMID | Study Design | Sample Size | Age: mean (SD) | Sex  M:F | Risk of Bias | Level of Evidence |
| (Lin et al., 2015) | 25780390 | Cross Sectional Study | 10 | Age of Experimental Group: 43.6 (10.8)  Age of Control Group: 45.2 (12.6) | Sex of Experimental Group: 5:0  Sex of Control Group: 5:0 | 7 | Level 4 |
| **Aim of Study:** To report the results of a pilot study, using the Localized COrrelated SpectroscopY (L-COSY) method, on chronic sports-induced repetitive head injury in elite athletes compared with healthy age-matched controls with no history of head injury.  **Key Outcome Measures:** MRI and MRS - the posterior cingulate gyrus was chosen for examination due to evidence that this area is sensitive to TBI in addition to this area being implicated in receiving higher tau deposition with CTE.  **Age at Head Injury:** Not Reported, **Years Since Head Injury:** Average of 17.4 (7.2) years of exposure to repetitive TBI in contact sports.  **Years of Education:** Not Reported; **Race (%):** Not Reported; **Population Type:** Athletes  **Diagnosis of Concussion:** Based on self-reported number of professional years and total number of years played in their sport as a proxy for RHI  **Key Findings:** None of the athletes showed structural MRI abnormalities using conventional imaging metrics. The variation of the method was calculated by repeated examination of a healthy control and phantom and found to be 10% and 5%, respectively, or less. The L-COSY measured large and statistically significant differences (p ≤ 0.05), between healthy controls and athletes with RHI. Men with RHI showed higher levels of glutamine/glutamate (31%), choline (65%), fucosylated molecules (60%) and phenylalanine (46%). The results were evaluated and the sample size of five found to achieve a significance level p = 0.05 and a power of 90%. Differences in N-acetyl aspartate and myo-inositol between RHI and controls were small and were not statistically significant. | | | | | | | |
| Lead Author and Study Year | PMID | Study Design | Sample Size | Age: mean (SD) | Sex  M:F | Risk of Bias | Level of Evidence |
| (Lipton et al., 2013) | 23757503 | Cross Sectional Study | 37 | Age of Experimental Group:  Low-heading group: 32.4 (6.2), Medium-heading group: 31.4 (5.5), and High-heading group: 28.7 (6.2) | Sex of Experimental Group: Low-heading group: 6:3, Medium-heading group: 15:4 and High-heading group: 8:1 | 10 | Level 4 |
| **Aim of Study:** To investigate the association of soccer heading with subclinical evidence of TBI.  **Key Outcome Measures:** Diffusion-tensor magnetic resonance (MR) imaging at 3T was performed (32 directions; b value, 800 sec/mm2; 2 3 2 3 2-mm voxels). Computerized battery of tests for cognitive functioning and questionnaire to quantify heading in the prior 12 months and lifetime concussion history.  **Age at Head Injury:** Not Reported, **Years Since Head Injury:** Not Reported. Estimated (>10) based on years of exposure to soccer: Low-heading group: 27 (6.1) years, Medium-heading group: 21.6 (7.9) years, and High-heading group: 19.9 (6.9) years  **Years of Education:** Low-heading group: 16.2 (1.6), Medium-heading group: 16.5 (1.5) and High-heading group: 15.3 (1.7); **Race (%):** Not Reported; **Population Type:** Athletes  **Diagnosis of Concussion:** Self-reported: subjects were asked to quantify heading in the prior 12 months and lifetime concussion exposure.  **Key Findings:** Participants had headed 32–5400 times (median, 432 times) over the previous year. Heading was associated with lower FA at three locations in temporo-occipital white matter with a threshold that varied according to location (885–1550 headings per year) (p < .00001). Lower levels of FA were also associated with poorer memory scores (p < .00001), with a threshold of 1800 headings per year. | | | | | | | |
| Lead Author and Study Year | PMID | Study Design | Sample Size | Age: mean (SD) | Sex  M:F | Risk of Bias | Level of Evidence |
| (Misquitta et al., 2018) | 29984163 | Cross Sectional Study | 399 | Age of Experimental Group: 55.6 (12.9)  Age of Control Group: Study controls: 50.8 (10) and Cam-CAN controls: 58.1 (16) | Sex of Experimental Group: 53:0  Sex of Control Group: Study controls: 25:0 and Cam-CAN controls: 321:0 | 10 | Level 4 |
| **Aim of Study:** To use structural segmentation and deformation-based morphometry (DBM) analyses to compare the effect of multiple concussions on regional brain volumes in retired professional athletes from the Canadian Football League (ex-CFL) with non-athlete control subjects with no history of concussion.  **Key Outcome Measures:** DBM analysis of subcortical structures using 3T MRI, APOE genotyping, the PAI, Rey Auditory Verbal Learning Test, and the Rey Visual Design Learning Test.  **Age at Head Injury:** Not Reported, **Years Since Head Injury:** Estimated (>10) based on number of years in CFL: average of 9 years, earlier in life.  **Years of Education:** 16.7 (1.7) for Ex-CFL players and 16 (1.9) for study controls; **Race (%):** Not Reported; **Population Type:** Athletes  **Diagnosis of Concussion:** Concussion exposure was based on players' recall of injury during a semi-structured interview in accordance with the Zurich Guidelines on Concussions (McCrory et al., 2013).  **Key Findings:** Volumetric analyses revealed greater hippocampal atrophy than expected for age in former athletes with multiple concussions than controls and smaller left hippocampal volume was associated with poorer verbal memory performance in the former athletes. DBM confirmed smaller bilateral hippocampal volume that was associated with poorer verbal memory performance in athletes. Number of years playing professional football was significantly related to smaller left and right hippocampus and left and right amygdala volumes, but not with ventricular volume. After Bonferroni correction for multiple comparisons, only correlations with the left and right amygdala remained significant (p < 0.01). There was no correlation between career years and self-reported number of concussions (r =-0.01, p = 0.93). | | | | | | | |
| Lead Author and Study Year | PMID | Study Design | Sample Size | Age: mean (SD) | Sex  M:F | Risk of Bias | Level of Evidence |
| (Monti et al., 2013) | 23986698 | Cross Sectional Study | 44 | Age of Experimental Group: young mTBI group: 22.4 (2), middle-aged mTBI group: 52.9 (9.4)  Age of Control Group: young control group: 22.3 (2.6), middle-aged control group: 52.5 (7.9) | Sex of Experimental Group: young mTBI group: 7:5, middle-aged mTBI group: 4:6  Sex of Control Group: young control group: 7:5, middle-aged control group: 4:6 | 10 | Level 4 |
| **Aim of Study:** To examine structural and functional brain aberrations in individuals who had sustained mTBI several decades ago in the brain regions and networks subserving relational memory.  **Key Outcome Measures:** fMRI study: 3T MRI was used. Subcortical volume analyses and functional analyses were done. Participants completed an event-related relational memory task during fMRI scanning where the goal was to form an association between a face and scene.  **Age at Head Injury:** Not Reported, **Years Since Head Injury:** young mTBI group: 4 (3.1), middle-aged mTBI group: 39 (12.6)  **Years of Education:** young mTBI group: 15.4 (0.52), middle-aged mTBI group: 17.3 (3.9), young control group: 15.6 (0.9), middle-aged control group: 17.3 (3.6); **Race (%):** Not Reported; **Population Type:** General Population  **Diagnosis of Concussion:** Only those participants whose mTBIs were diagnosed by a medical professional and/or resulted in LOC for less than 30 minutes were included in the mTBI groups; based on criteria from the Mild Traumatic Brain Inury Committee (1993) at the American Congress of Rehabilitation Medicine.  **Key Findings:** Results indicated that middle-aged adults with a head injury in their remote past had impaired memory compared to sex-, age-, and education- matched control participants. The present study demonstrated that these individuals also had smaller bilateral hippocampi, and had reduced neural activity during memory performance in cortical regions important for memory retrieval. Reduced neural activity in multiple regions of the PFC was found for the MI group relative to the MC group, including the right inferior frontal gyrus, right medial PFC (mPFC), bilateral frontopolar cortex, and middle and superior frontal gyri. | | | | | | | |
| Lead Author and Study Year | PMID | Study Design | Sample Size | Age: mean (SD) | Sex  M:F | Risk of Bias | Level of Evidence |
| (Multani et al., 2016) | 27142715 | Cross Sectional Study | 35 | Age of Experimental Group: 49.6 (12)  Age of Control Group: 46.7 (10) | Sex of Experimental Group: 18:0  Sex of Control Group: 17:0 | 10 | Level 4 |
| **Aim of Study:** To examine the effect of multiple concussions on whole-brain white-matter integrity in retired professional football players.  **Key Outcome Measures:** DTI: Whole brain tract-based spatial statistics analysis were performed to examine white matter tract integrity (tractography on the SLF was performed using the probabilistic tractography [probtrackx] in FSL) and to compare findings with neuropsychological outcomes. Assessment measures: PAI, RDVLT, and WTAR.  **Age at Head Injury:** Not Reported, **Years Since Head Injury:** 16.2 (13)  **Years of Education:** Players: 17.3 (2), controls: 16.4 (2); **Race (%):** Not Reported; **Population Type:** Athletes  **Diagnosis of Concussion:** The history of concussions was determined by the player’s ability to recall injuries caused by a blow to the head or body that resulted in concussion symptoms, including at least one of the following: headache, nausea, vomiting, dizziness/balance problems, fatigue, trouble sleeping, drowsiness, sensitivity to light or noise, blurred vision, difficulty remembering, and trouble concentrating.  **Key Findings:** Whole brain tract-based spatial statistics analysis revealed increased AD in the right hemisphere of retired players in the (1) superior longitudinal fasciculus (SLF), (2) corticospinal tract, and (3) anterior thalamic radiations, suggesting chronic axonal degeneration in these tracts. TBSS results were not significantly different for FA, RD, and MD between retired players and controls. Moreover, retired players reported significantly higher neuropsychiatric and cognitive symptoms than controls, and worsening symptoms since their last concussion. | | | | | | | |
| Lead Author and Study Year | PMID | Study Design | Sample Size | Age: mean (SD) | Sex  M:F | Risk of Bias | Level of Evidence |
| (Rajesh et al., 2017) | 28726543 | Case Control Study | 44 | Age of Experimental Group: 20-65 years post-injury group: 52.9 (9.4) and 1-10 years post-injury group: 22.42 (2.02).  Age of Control Group: 20-65 years controls: 52.5 (7.86) and 1-10 years controls: 22.55 (2.58). | Sex of Experimental Group: 20-65 years post-injury group: 6:4 and 1-10 years post-injury group: 5:7  Sex of Control Group: 20-65 years controls: 6:4 and 1-10 years controls: 5:7 | 9 | Level 4 |
| **Aim of Study:** To examine two groups of cognitively resolved remote-mTBI individuals with 1-10 years or 20-65 years time post-injury.  **Key Outcome Measures:** Examined resting state functional connectivity and structure (volumetric and thickness-based morphometry, and FA).  **Age at Head Injury:** Not Reported, **Years Since Head Injury:** 20-65 years group: 39 (12.57), 1-10 years group: 4 (3.19)  **Years of Education:** 20-65 years post-injury group: 17.3 (3.86), 1-10 years post-injury group: 15.42 (0.51), 20-65 years age-matched control group: 17.3 (3.6) and 1-10 years age-matched control group: 15.73 (0.79); **Race (%):** Not Reported; **Population Type:** General Population  **Diagnosis of Concussion:** Diagnosis of mTBI was made by a medical professional and/or those who had LOC <30 min and/or who had a post-traumatic amnesia <24 h.  **Key Findings:** Abnormalities in brain architecture were only found in the TBI 1-10 years post-injury group and were characterized by functional hypoactivation in the right frontal pole, smaller superior frontal gyrus and frontal pole volume, and less FA in the genu of the corpus callosum that extended near the right frontal pole compared with matched controls. For the resting state DMN data, only the comparison for Control > mTBI 1-10 years post-injury showed significant differences, and only for the seed placed in the PCC. Specifically, the PCC region in healthy controls showed significantly greater correlation with the right frontal pole/anterior prefrontal cortex relative to mTBI 1-10 years post-injury group (p < 0.05 cluster corrected). In secondary analysis within the combined group of mTBI (n = 22), age at last injury did not correlate with magnitude of functional activation n the right frontal pole (r = -0.20; p = 0.38). | | | | | | | |
| Lead Author and Study Year | PMID | Study Design | Sample Size | Age: mean (SD) | Sex  M:F | Risk of Bias | Level of Evidence |
| (Rowland et al., 2018) | 29634322 | Cross Sectional Study | 16 | Age of Experimental Group: PTSD group: 34.9 (4.7)  Age of Control Group: No PTSD group: 43.4 (7.8) | Sex of Experimental Group: 7:0  Sex of Control Group: 9:0 | 12 | Level 4 |
| **Aim of Study:** To determine differences in whole-brain resting-state functional networks associated with the development of PTSD following deployment-acquired mild TBI.  **Key Outcome Measures:** Graph theory metrics, including small-worldness, clustering coefficient, and modularity, were calculated from individually constructed whole-brain networks based on 5-min eyes-open resting-state magnetoencephalography (MEG) recordings. Tests: the Structured Clinical Interview for DSM-IV Diagnosis, Clinician-Administered PTSD Scale-5, Test of Premorbid Function, neurobehavioral symptom inventory (NSI), and PTSD Checklist-5 (PCL-5).  **Age at Head Injury:** Not Reported, **Years Since Head Injury:** PTSD group: 9.07 (3.77) years and no PTSD group: 13.1 (7.7)  **Years of Education:** PTSD group: 15.1 (1.6) years and no PTSD group: 16.6 (2.2); **Race (%):** 14.3% minorities in the PTSD group and 28.6% in the no PTSD group; **Population Type:** Veterans  **Diagnosis of Concussion:** A structured clinician-administered interview was used to determine mTBI history according to the American Congress of Rehabilitation Medicine criteria (Menon, Schwab, Wright, & Maas, 2010). Severity was based on VA/DoD consensus criteria (2009), with mTBI displaying LOC <30 min, alteration of consciousness (AOC) < 24 h, and/or post traumatic amnesia (PTA) <24 h.  **Key Findings:** Results demonstrated that participants with current PTSD displayed higher levels of small-worldness, F(1,12) = 5.364, p < 0.039, partial eta squared = 0.309, and Cohen’s d = 0.972, and clustering coefficient, F(1, 12) = 12.204, p < 0.004, partial eta squared = 0.504, and Cohen’s d = 0.905, than those without PTSD. There were no between-group differences in modularity or the number of modules present. | | | | | | | |
| Lead Author and Study Year | PMID | Study Design | Sample Size | Age: mean (SD) | Sex  M:F | Risk of Bias | Level of Evidence |
| (Small et al., 2013) | 23343487 | Cross Sectional Study | 10 | Age of Experimental Group: median age: 59, Range: 45-73  Age of Control Group: median age: 60, Range: 45-66 | Sex of Experimental Group: 5:0  Sex of Control Group: 5:0 | 10 | Level 4 |
| **Aim of Study:** To perform PET scans after intravenous injections of FDDNP and explore whether brain tau deposits can be detected in a small group of retired NFL players with cognitive and mood symptoms in comparison with a group of male controls of comparable age, educational achievement, and body mass index (BMI).  **Key Outcome Measures:** Neuropsychiatric evaluations and FDDNP-PET. PET signals in subcortical (caudate, putamen, thalamus, subthalamus, midbrain, cerebellar white matter) and cortical (amygdala, frontal, parietal, posterior cingulate, medial and lateral temporal) regions were examined. Subjects had screening laboratory tests and structural imaging scans (computed tomography [CT] or magnetic resonance imaging) to rule out other causes of mental symptoms (e.g., stroke, tumor) and for co-registration with PET scans for region-of-interest (ROI) analyses. The MMSE, Hamilton Rating Scale for Depression (HAM-D), and neuropsychological tests were administered to confirm diagnoses.  **Age at Head Injury:** Not Reported, **Years Since Head Injury:** Not Reported, but the athletes all played between 10 - 16 years (median: 14).  **Years of Education:** Players**:** median years of education: 17, controls: 15; **Race (%):** 4 white, 1 African American; **Population Type:** Athletes  **Diagnosis of Concussion:** Not described  **Key Findings:** FDDNP signals were higher in players (median BMI = 32) compared with controls (median BMI = 34) in all subcortical regions and the amygdala, areas that produce tau deposits following trauma. Players had significantly higher FDDNP signals compared with controls in caudate (median levels:1.48 versus 1.23, p = 0.03), putamen (1.47 versus 1.20, p = 0.05), thalamus (1.48 versus 1.29, p=0.03), subthalamus (1.45 versus 1.25, p = 0.03) midbrain (1.31 versus 1.14, p = 0.03), and cerebellar white matter (1.15 versus 1.09, p = 0.05) regions. The two groups did not differ significantly in FDDNP binding in cortical regions except for the amygdala (1.30 versus 1.14, p = 0.03). Although none of the Spearman correlations reached statistical significance (as expected due to the small sample size), the plots show an increase in FDDNP binding levels with increase in number of concussions. | | | | | | | |
| Lead Author and Study Year | PMID | Study Design | Sample Size | Age: mean (SD) | Sex  M:F | Risk of Bias | Level of Evidence |
| (Tremblay et al., 2013) | 22581847 | Cross Sectional Study | 30 | Age of Experimental Group: 60.87 (7.51)  Age of Control Group: 58.13 (5.28) | Sex of Experimental Group: 15:0  Sex of Control Group: 15:0 | 10 | Level 4 |
| **Aim of Study:** To document possible structural anomalies of brain tissue in vivo in retired athletes who sustained sports concussions in early adulthood and to establish neurocognitive links between hypothesized structural and neurometabolic anomalies in retired concussed athletes with cognitive decline.  **Key Outcome Measures:** 3T MRI (VBM for hippocampal volume and corical thickness): 1H MR spectra were obtained from the voxels localized in the bilateral medial temporal lobes and bilateral prefrontal cortices. The following metabolites were quantified: N-acetylaspartate (NAA), myo-inositol (mI), choline-containing compounds (Cho), as well as H_2_O for an internal reference. Genotyping was done and cognitive measures included: the MMSE, BDI-II, Taylor complex figure test (TCFT), RAVLT, verbal fluency, TMT A and B, and SDMT.  **Age at Head Injury:** Sustained their last sports concussion in early adulthood 24 (4.55), **Years Since Head Injury:** 37.08 (7.10)  **Years of Education:** controls: 17.27 (3.45) and concussion history group: 16.67 (4.06); **Race (%):** Not Reported; **Population Type:** Athletes  **Diagnosis of Concussion:** A standardized concussion history questionnaire was administered in an interview with a sports physician. Concussion was defined according to the 2009 Consensus Statement on Concussion in Sports (McCrory et al., 2009).  **Key Findings:** Compared to controls, former athletes exhibited: 1) Abnormal enlargement of the lateral ventricles, 2) cortical thinning in regions more vulnerable to the aging process, 3) various neurometabolic anomalies found across regions of interest, 4) episodic memory and verbal fluency decline that correlated with neuroimaging findings in concussed participants. 1H MRS examination detected a significant elevation of mI/H_2_O in the left medial temporal lobe of formerly concussed participants that correlated strongly with the TCFT delayed recall score, after FDR correction for multiple comparisons. The same ROI also exhibited an abnormal reduction in Cho, while the right prefrontal cortex presented a significant increase in the same metabolite, when referenced to H_2_O. When the age variable was introduced in addition to the group, lateral ventricular volume presented a significant age by group interaction, suggesting that ventricular volume expansion was further exacerbated with the advancing age in the group with a history of concussion. | | | | | | | |
| Lead Author and Study Year | PMID | Study Design | Sample Size | Age: mean (SD) | Sex  M:F | Risk of Bias | Level of Evidence |
| (Tremblay et al., 2019) | 31233955 | Cross Sectional Study | 74 | Age of Experimental Group: Recent mTBI: 62.89 (4.92), remote mTBI: 60.87 (7.51)  Age of Control Group: Controls for recent mTBI: 61.76 (6.6), controls for remote mTBI: 58.13 (5.28) | Sex of Experimental Group: Recent mTBI group: 7:12 and remote mTBI group: 15:0  Sex of Control Group: Controls for recent mTBI group: 11:14 and controls for remote mTBI group: 15:0 | 8 | Level 4 |
| **Aim of Study:** To determine whether patients who sustain a mTBI earlier in life fare better than patients who sustain a mTBI at an older age.  **Key Outcome Measures:** 3T MRI (DWI): Deformation-based morphometry (DBM) provides an estimate of volume changes across the entire T1-weigthed brain image for assessment of white and grey matter atrophy. Tract-based spatial statistics (TBSS) was done to compare various DWI metrics across groups to localise brain changes in diffusion at a voxel-wise level.  **Age at Head Injury:** Recent mTBI group: 61.05 (4.9) and remote mTBI group: 24.6 (6.34), **Years Since Head Injury:** Recent group (in months): 22.06 (13.12) and remote group (in months): 443.20 (77.30)  **Years of Education:** Recent mTBI group: 16.67 (1.97) and remote mTBI group: 16.67 (4.06). Controls for recent mTBI group: 16.92 (2.25) and controls for remote mTBI group: 17.27 (3.45); **Race (%):** The entire sample was 100% Caucasian; **Population Type:** Other: Athletes for remote group and general population for recent group.  **Diagnosis of Concussion:** A standardized concussion history questionnaire was administered in an interview setting by an experienced sports physician. A concussion was defined according to the 2009 Consensus Statement on Concussion in Sports (McCrory et al., 2009).  **Key Findings:** Results showed a significant interaction on DWI measures indicating larger anomalies in adults who sustained a mTBI at a younger age (F1,70, p < .05, FDR corrected). Total brain volume did not differ between mTBI and control participants in the recent and remote cohort. Analyses of MD images revealed a significant Group by Cohort interaction effect following correction for multiple comparisons. Regions affected included primarily the anterior aspect of the corpus callosum and frontal white matter. Decomposition of the interaction term into simple effects revealed large areas of increased MD in patients from the remote mTBI cohort relative to controls. | | | | | | | |
| Lead Author and Study Year | PMID | Study Design | Sample Size | Age: mean (SD) | Sex  M:F | Risk of Bias | Level of Evidence |
| (Vasilevskaya et al., 2020) | 32097865 | Cross Sectional Study | 38 | Age of Experimental Group: 55.08 (13.39)  Age of Control Group: 51.36 (14.44) | Sex of Experimental Group: 37:1 (for the whole sample; n = 13 for APOE4 group and n = 25 for APOE4 non-carriers) | 10 | Level 4 |
| **Aim of Study:** To examine the effect of the APOE4 allele and MAPTH1H1 on SUVRs of PET tau-specific [F-18]AV-1451 tracer in former professional contact sport athletes (CFL) at risk for CTE.  **Key Outcome Measures:** PET tau imaging with 5mCi of [F-18]AV-1451 tracer was performed (imaged using 3T MRI). A sandwich ELISA method was used to measure Aβ42, phosphorylated tau (p-tau) and total tau (t-tau) levels in CSF. Blood was collected from all participants and genomic DNA was extracted using a Qiagen kit from whole blood. The APOE genotypes and MAPT haplotypes were determined. Cognitive tests used: TMT A and B, RAVLT, RVDLT, SDMT, PAI, digit span forward and backward.  **Age at Head Injury:** Not Reported, **Years Since Head Injury:** APOE4 Carriers: 19.58 (21.59) and APOE4 Non-Carriers: 21.59 (15.82)  **Years of Education:** APOE4 Carriers: 15.92 (1.61), APOE4 Non-Carriers: 15.36 (1.73); **Race (%):** Not Reported; **Population Type:** Athletes  **Diagnosis of Concussion:** Concussion exposure was determined based on the player's recall of injury using the concussion definition provided by the Concussion in Sport Group (Meeuwisse et al., 2017). All players had a semi-structured interview to verify information and to jog memory.  **Key Findings:** Cortical grey matter PET tau SUVR values were significantly higher in APOE4 carriers compared to non-carriers (p= 0.020). There was no significant difference in SUVR between MAPTH1H1 vs non-H1H1 carrier genes (p = 1.00). There was a significantly higher APOE4 allele frequency in the high cortical grey matter PET tau group, compared to the low cortical grey matter PET tau group (p = 0.048). | | | | | | | |
| Lead Author and Study Year | PMID | Study Design | Sample Size | Age: mean (SD) | Sex  M:F | Risk of Bias | Level of Evidence |
| (Wang, Wei, Yu, Li, & Li, 2017) | 28689050 | Cross Sectional Study | 90 | Age of Experimental Group: mTBI group: 67.63 (6.65), preclinical mTBI: 73.5 (6.2), MCI to AD mTBI: 73.65 (6.86), and AD mTBI: 72.8 (8.31)  Age of Control Group: mTBI controls: 68.63 (5.26), preclinical controls: 72.7 (4.81), MCI to AD controls: 72.18 (6.12), and AD controls: 72.3 (10.32) | Sex of Experimental Group: mTBI group: 4:4, preclinical mTBI: 6:4, MCI to AD mTBI: 15:2, and AD dementia mTBI: 6:4  Sex of Control Group: mTBI controls: 4:4, preclinical controls: 6:4, MCI to AD controls: 15:2, and AD dementia controls: 6:4 | 11 | Level 4 |
| **Aim of Study:** To investigate whether self-reported mTBI is associated with decreased AD-vulnerable cortical thickness, and to assess the relationship between AD-vulnerable cortical thickness and AD-related biomarker in ADNI subjects.  **Key Outcome Measures:** 3T Structural MRI, 18FAV45 PET, and cerebrospinal fluid (CSF) data. Cortical thickness of eight AD-vulnerable regions, mean AD-vulnerable cortical thickness, 18F-AV45 PET mean amyloid SUVR, CSF Aβ42, CSF total tau (T-tau), and CSF phosphorylated tau (P-tau) were compared between mTBI and non-TBI groups.  **Age at Head Injury:** normal mTBI group: 29.88 (23.2), preclinical mTBI group: 30 (23.66), MCI to AD mTBI group: 37.1 (27.35), and AD dementia mTBI group: 36.4 (21.37), **Years Since Head Injury:** Not reported  **Years of Education:** normal mTBI group: 17.5 (1.69), preclinical mTBI group: 17.2 (2.35), MCI to AD mTBI group: 17 (3.04), AD dementia mTBI group: 15.6 (2.27), normal control group: 17.13 (3.04), preclinical control group: 17 (2.11), MCI to AD control group: 16.94 (2.84), and AD dementia control group: 16.1 (1.97); **Race (%):** Not Reported; **Population Type:** General Population  **Diagnosis of Concussion:** Identified 45 mild closed head injury subjects from the ADNI cohort according to the Mayo Clinic TBI standards.  **Key Findings:** Of the 45 subjects, eight subjects were healthy; ten were preclinical AD; seventeen had MCI due to AD; ten had AD. Preclinical AD subjects with self-reported mTBI had decreased cortical thickness in mean (p = 0.038) and three AD-vulnerable cortical regions than non-TBI subjects (p < 0.05). The mean cortical thickness of all identified AD-vulnerable regions was correlated with CSF T-tau (r = -0.81, p = 0.001). The regions included when examining mean differences between groups were: superior parietal cortex, inferior parietal cortex, middle temporal gyrus, inferior temporal gyrus, precuneus, posterior cingulate cortex, entorhinal cortex, and temporal pole. The three individual regions that were significant in this study were the inferior parietal cortex (p = 0.005), superior parietal cortex (p = 0.009), and precuneus (p = 0.015). The CSF P-tau was also higher in the preclinical AD with self-reported mTBI group (p = 0.028). Among all the preclinical AD subjects, the mean AD-vulnerable cortical thickness was correlated with CSF T-Tau (r = -0.55, p = 0.018) and CSF P-Tau (r = -0.60, p = 0.007). There was no statistical difference in the comparison of normal, MCI due to AD, and AD groups. | | | | | | | |
| Lead Author and Study Year | PMID | Study Design | Sample Size | Age: mean (SD) | Sex  M:F | Risk of Bias | Level of Evidence |
| (Ware et al., 2020) | 30565025 | Cross Sectional Study | 19 | Age of Experimental Group: 45.7 (9.71)  Age of Control Group: 43.4 (9.11) | Sex of Experimental Group: 10:0  Sex of Control Group: 9:0 | 10 | Level 4 |
| **Aim of Study:** To determine the specificity of microstructural changes in subregions of the corpus callosum in chronic, repetitive boxing-related TBI relative to noncontact sport athlete controls. The second aim was to identify any group differences on commonly reported psychiatric and neuropsychological difficulties. For the third aim, the clinical implications of white matter microstructural findings were examined.  **Key Outcome Measures:** Deterministic DTI of different regions of the corpus callosum. Anxiety using the BAI, depression using the Centre for Epidemiological Studies-Depression, executive function (TMT), and fine motor dexterity using the Grooved Pegboard.  **Age at Head Injury:** Not Reported, **Years Since Head Injury:** Not Reported. Estimated (>10) based on ages they started boxing: 10 (4.5)  **Years of Education:** Experimental group: 13 (1.49), control group: 13.78 (1.92); **Race (%):** Only Hispanic % was given: experimental group: 40%, control group: 22%; **Population Type:** Athletes  **Diagnosis of Concussion:** Not described, however history of boxing was used as a proxy for RHI. In addition, number of professional bouts, years sparring and professional knockouts with and without loss of consciousness were collected.  **Key Findings:** Quantitative DTI metrics were estimated for corpus callosum subregions. Group by Region interaction effects were observed on FA (η2p ≥ .21). Follow-up indicated large effects of group (η2p ≥ .26) on splenium FA (boxers<comparisons) and genu MD (boxers > comparisons), but not RD. In boxers, years sparring, professional bouts, and knockout history correlated strongly (r > .40) with DTI metrics and fine motor dexterity. A similar pattern was observed between total number of professional knockouts (with and without associated LOC) and diffusion metrics in the body of the corpus callosum; greater number of professional knockouts was strongly associated with lower FA and higher MD and RD values in the body of the corpus callosum, respectively. Number of professional knockouts with LOC was strongly, negatively correlated with FA in splenium of the corpus callosum; greater number of knockouts with LOC was strongly associated with lower FA values in the splenium of the corpus callosum. | | | | | | | |
| Lead Author and Study Year | PMID | Study Design | Sample Size | Age: mean (SD) | Sex  M:F | Risk of Bias | Level of Evidence |
| (Wilde et al., 2016) | 26414735 | Cross Sectional Study | 19 | Age of Experimental Group: 45.7 (9.71)  Age of Control Group: 43.4 (9.11) | Sex of Experimental Group: 10:0  Sex of Control Group: 9:0 | 9 | Level 4 |
| **Aim of Study:** To evaluate imaging and cognitive indications of neurodegeneration after RHI and mTBI associated with extended exposure to boxing.  **Key Outcome Measures:** 3T MRI: DTI, particularly bilateral uncinate fasciculus; inferior longitudinal fasciculus; ventral striatum; and cerebral peduncle. Cognitive measures: verbal selective reminding test and the serial reaction time test.  **Age at Head Injury:** Not Reported, **Years Since Head Injury:** Not Reported. Estimated (>10) based on the average number of years boxing which was 35.70 (9.15), indicating that participants began boxing early in life.  **Years of Education:** Experimental group: 13.00 (1.49); Controls: 13.78 (1.92); **Race (%):** Not Reported; **Population Type:** Athletes  **Diagnosis of Concussion:** Not described, however they asked about knockouts during fights (M = 0.89, SD = 0.60) including knockouts with loss of consciousness (M = 0.67, SD = 0.71).  **Key Findings:** Evans Index (maximum width of the anterior horns of the lateral ventricles/maximal width of the internal diameter of the skull) was significantly larger in the boxers (F = 4.52; p = 0.050; Cohen’s f = 0.531). FA and the apparent diffusion coefficient (ADC) measured by tractography did not significantly differ between groups. Years of boxing had the most consistent, negative correlations with FA, ranging from -0.65 for the right ventral striatum to -0.92 for the right cerebral peduncle. Years of boxing was negatively related to the number of words consistently recalled over trials (r = -0.74; p = 0.02), delayed recall (r = -0.83; p = 0.003), and serial RT (r = 0.66; p = 0.05). Of the measures of exposure, years of boxing had the most significant, negative correlations with FA, including for the right uncinate fasciculus (r = -0.807; p = 0.026) and the right cerebral peduncle (r = -0.827; p = 0.022). Convergent with the correlations for FA, years of boxing was positively related to ADC, reaching significance for the left inferior longitudinal fasciculus (r = 0.798; p = 0.032) and left uncinate fasciculus (r = -0.763; p = 0.046). | | | | | | | |
| Lead Author and Study Year | PMID | Study Design | Sample Size | Age: mean (SD) | Sex  M:F | Risk of Bias | Level of Evidence |
| (Zivadinov et al., 2018) | 30080799 | Cross Sectional Study | 42 | Age of Experimental Group: 56.7 (9.5)  Age of Control Group: 55.4 (9.3) | Sex of Experimental Group: 21:0  Sex of Control Group: 21:0 | 10 | Level 4 |
| **Aim of Study:** To investigate multimodal metabolic and structural brain MRI differences in retired professional contact sport athletes compared with noncontact sport athletes.  **Key Outcome Measures:** 3T MRI (structural and functional analyses); white matter signal abnormalities; global and regional atrophy measures; DTI measures; cerebral microbleeds; quantitative susceptibility mapping; MR spectroscopy measures; PWI measures.  **Age at Head Injury:** Not Reported. Athletes were NFL players and NHL players who entered into their respective leagues in their 20s, **Years Since Head Injury:** Not Reported  **Years of Education:** Experimental group: high school = 36.4%, some college = 18.2%, college degree = 31.8%, associate's degree = 9.1%, postgraduate degree = 4.5%; Control group: high school = 9.5%, some college = 0%, college degree = 42.9%, associate's degree = 9.5%, postgraduate degree = 38.1%; **Race (%):** Experimental = 100% Caucasian; Controls = 86.4% Caucasian, 13.6% African American; **Population Type:** Athletes (retired NFL and NHL athletes)  **Diagnosis of Concussion:** Not Reported. Contact sport play used as proxy for RHI.  **Key Findings:** No significant differences were found for structural or functional MRI measures between retired contact sport athletes and noncontact sport athletes. This multimodal imaging study did not show any microstructural, metabolic brain tissue injury differences in retired contact versus non-contact sport athletes. | | | | | | | |

# **Supplementary Table 2. Downs and Black checklist for the assessment of the methodological quality of both randomized and non-randomized studies.**

| Quality Assessment | Reporting | | | | | | | | | | External Validity | | | Internal Validity-Bias | | | | | | | Internal Validity Confounding-Selection Bias | | | | | | Power |  |
| --- | --- | --- | --- | --- | --- | --- | --- | --- | --- | --- | --- | --- | --- | --- | --- | --- | --- | --- | --- | --- | --- | --- | --- | --- | --- | --- | --- | --- |
|  | 1 | 2 | 3 | 4 | 5 | 6 | 7 | 8 | 9 | 10 | 11 | 12 | 13 | 14 | 15 | 16 | 17 | 18 | 19 | 20 | 21 | 22 | 23 | 24 | 25 | 26 | 27 | Total |
| Bryant et al., 2020 | 1 | 1 | 1 | 0 | 0 | 1 | 1 | 0 | 1 | 1 | 0 | 0 | 0 | 0 | 0 | 1 | 0 | 1 | 0 | 1 | 0 | 0 | 0 | 0 | 0 | 0 | - | 10 |
| Casson et al., 2014 | 1 | 1 | 1 | 0 | 1 | 1 | 0 | 0 | 0 | 0 | 0 | 0 | 0 | 0 | 0 | 0 | 1 | 0 | 0 | 1 | 0 | 1 | 0 | 0 | 0 | 0 | - | 8 |
| Chong et al., 2018 | 1 | 1 | 1 | 0 | 1 | 1 | 1 | 0 | 0 | 1 | 0 | 0 | 0 | 0 | 0 | 0 | 0 | 1 | 0 | 1 | 0 | 0 | 0 | 0 | 0 | 0 | - | 9 |
| Clark et al., 2018 | 1 | 1 | 1 | 0 | 1 | 1 | 1 | 0 | 0 | 1 | 1 | 1 | 0 | 0 | 0 | 1 | 0 | 1 | 0 | 1 | 1 | 1 | 0 | 0 | 0 | 0 | - | 14 |
| De Beaumont et al., 2013 | 1 | 1 | 1 | 0 | 1 | 1 | 1 | 0 | 0 | 1 | 0 | 0 | 0 | 0 | 0 | 1 | 0 | 1 | 0 | 1 | 0 | 0 | 0 | 0 | 0 | 0 | - | 10 |
| Ford et al., 2013 | 1 | 1 | 1 | 0 | 1 | 1 | 1 | 0 | 0 | 1 | 0 | 0 | 0 | 0 | 0 | 1 | 0 | 1 | 0 | 1 | 1 | 0 | 0 | 0 | 0 | 0 | - | 11 |
| Gardner et al., 2016 | 1 | 1 | 1 | 0 | 1 | 1 | 1 | 0 | 0 | 1 | 0 | 0 | 0 | 0 | 0 | 1 | 0 | 1 | 0 | 1 | 1 | 1 | 0 | 0 | 1 | 0 | - | 13 |
| Gilmore et al., 2020 | 1 | 1 | 1 | 0 | 1 | 1 | 1 | 0 | 0 | 1 | 0 | 0 | 0 | 0 | 0 | 1 | 0 | 1 | 0 | 1 | 0 | 0 | 0 | 0 | 0 | 0 | - | 10 |
| Goswami et al., 2016 | 1 | 1 | 1 | 0 | 1 | 1 | 0 | 0 | 0 | 1 | 0 | 0 | 0 | 0 | 0 | 1 | 0 | 1 | 0 | 1 | 0 | 0 | 0 | 0 | 0 | 0 | - | 9 |
| Haglund et al., 1990 | 1 | 1 | 1 | 0 | 0 | 1 | 0 | 0 | 0 | 0 | 1 | 0 | 0 | 0 | 0 | 1 | 0 | 1 | 0 | 1 | 0 | 0 | 0 | 0 | 0 | 0 | - | 8 |
| Hampshire et al., 2013 | 1 | 0 | 0 | 0 | 0 | 1 | 0 | 0 | 0 | 1 | 0 | 0 | 0 | 0 | 0 | 1 | 0 | 1 | 0 | 1 | 0 | 0 | 0 | 0 | 0 | 0 | - | 6 |
| Hart et al., 2013 | 1 | 1 | 1 | 0 | 1 | 1 | 0 | 0 | 0 | 1 | 0 | 0 | 0 | 0 | 0 | 1 | 0 | 1 | 0 | 1 | 0 | 0 | 0 | 0 | 0 | 0 | - | 9 |
| June et al., 2020 | 1 | 1 | 1 | 0 | 1 | 1 | 1 | 0 | 0 | 1 | 0 | 0 | 0 | 0 | 0 | 1 | 0 | 1 | 0 | 1 | 1 | 0 | 0 | 0 | 0 | 0 | - | 11 |
| Kelman et al., 2020 | 1 | 1 | 1 | 0 | 0 | 1 | 1 | 0 | 0 | 1 | 0 | 0 | 0 | 0 | 0 | 1 | 0 | 1 | 0 | 1 | 0 | 0 | 0 | 0 | 0 | 0 | - | 9 |
| Koerte et al., 2015 | 1 | 1 | 1 | 0 | 1 | 1 | 1 | 0 | 0 | 1 | 0 | 0 | 0 | 0 | 0 | 1 | 0 | 1 | 0 | 1 | 0 | 0 | 0 | 0 | 0 | 0 | - | 10 |
| Koerte et al., 2016 | 1 | 1 | 1 | 0 | 0 | 1 | 1 | 0 | 0 | 1 | 0 | 0 | 0 | 0 | 0 | 1 | 0 | 1 | 0 | 1 | 0 | 1 | 0 | 0 | 0 | 0 | - | 10 |
| Koerte et al., 2016 | 1 | 1 | 1 | 0 | 1 | 1 | 1 | 0 | 0 | 1 | 0 | 0 | 0 | 0 | 0 | 1 | 0 | 1 | 0 | 1 | 0 | 0 | 0 | 0 | 0 | 0 | - | 10 |
| Kuhn et al., 2016 | 1 | 1 | 1 | 0 | 1 | 1 | 1 | 0 | 0 | 1 | 0 | 0 | 0 | 0 | 0 | 1 | 0 | 1 | 0 | 1 | 0 | 0 | 0 | 0 | 0 | 0 | - | 10 |
| Lepage et al., 2019 | 1 | 1 | 1 | 0 | 1 | 1 | 1 | 0 | 0 | 1 | 0 | 0 | 0 | 0 | 0 | 1 | 0 | 1 | 0 | 1 | 0 | 0 | 0 | 0 | 0 | 0 | - | 10 |
| Lin et al., 2015 | 1 | 1 | 1 | 0 | 0 | 1 | 1 | 0 | 0 | 0 | 0 | 0 | 0 | 0 | 0 | 1 | 0 | 1 | 0 | 0 | 0 | 0 | 0 | 0 | 0 | 0 | - | 7 |
| Lipton et al., 2013 | 1 | 1 | 1 | 0 | 1 | 1 | 1 | 0 | 0 | 1 | 0 | 0 | 0 | 0 | 0 | 1 | 0 | 1 | 0 | 1 | 0 | 0 | 0 | 0 | 0 | 0 | - | 10 |
| Misquitta et al., 2018 | 1 | 1 | 1 | 0 | 1 | 1 | 1 | 0 | 0 | 1 | 0 | 0 | 0 | 0 | 0 | 1 | 0 | 1 | 0 | 1 | 0 | 0 | 0 | 0 | 0 | 0 | - | 10 |
| Monti et al., 2013 | 1 | 1 | 1 | 0 | 1 | 1 | 1 | 0 | 0 | 1 | 0 | 0 | 0 | 0 | 0 | 1 | 0 | 1 | 0 | 1 | 0 | 0 | 0 | 0 | 0 | 0 | - | 10 |
| Multani et al., 2016 | 1 | 1 | 1 | 0 | 1 | 1 | 1 | 0 | 0 | 1 | 0 | 0 | 0 | 0 | 0 | 1 | 0 | 1 | 0 | 1 | 0 | 0 | 0 | 0 | 0 | 0 | - | 10 |
| Rajesh et al., 2017 | 1 | 1 | 1 | 0 | 1 | 1 | 0 | 0 | 0 | 1 | 0 | 0 | 0 | 0 | 0 | 1 | 0 | 1 | 0 | 1 | 0 | 0 | 0 | 0 | 0 | 0 | - | 9 |
| Rowland et al., 2018 | 1 | 1 | 1 | 0 | 1 | 1 | 1 | 0 | 0 | 1 | 0 | 0 | 0 | 0 | 0 | 1 | 1 | 1 | 0 | 1 | 1 | 0 | 0 | 0 | 0 | 0 | - | 12 |
| Small et al., 2013 | 1 | 1 | 1 | 0 | 1 | 1 | 1 | 0 | 0 | 1 | 0 | 0 | 0 | 0 | 0 | 1 | 0 | 1 | 0 | 1 | 0 | 0 | 0 | 0 | 0 | 0 | - | 10 |
| Tremblay et al., 2013 | 1 | 1 | 1 | 0 | 1 | 1 | 1 | 0 | 0 | 1 | 0 | 0 | 0 | 0 | 0 | 1 | 0 | 1 | 0 | 1 | 0 | 0 | 0 | 0 | 0 | 0 | - | 10 |
| Tremblay et al., 2019 | 1 | 1 | 1 | 0 | 1 | 1 | 0 | 0 | 0 | 0 | 0 | 0 | 0 | 0 | 0 | 1 | 0 | 1 | 0 | 1 | 0 | 0 | 0 | 0 | 0 | 0 | - | 8 |
| Vasilevskaya et al., 2020 | 1 | 1 | 1 | 0 | 1 | 1 | 1 | 0 | 0 | 1 | 0 | 0 | 0 | 0 | 0 | 1 | 0 | 1 | 0 | 1 | 0 | 0 | 0 | 0 | 0 | 0 | - | 10 |
| Wang et al., 2017 | 1 | 1 | 1 | 0 | 1 | 1 | 1 | 0 | 0 | 1 | 0 | 0 | 0 | 0 | 0 | 1 | 0 | 1 | 0 | 1 | 1 | 0 | 0 | 0 | 0 | 0 | - | 11 |
| Ware et al., 2020 | 1 | 1 | 1 | 0 | 1 | 1 | 1 | 0 | 0 | 1 | 0 | 0 | 0 | 0 | 0 | 1 | 0 | 1 | 0 | 1 | 0 | 0 | 0 | 0 | 0 | 0 | - | 10 |
| Wilde et al., 2016 | 1 | 1 | 1 | 0 | 0 | 1 | 1 | 0 | 0 | 1 | 0 | 0 | 0 | 0 | 0 | 1 | 0 | 1 | 0 | 1 | 0 | 0 | 0 | 0 | 0 | 0 | - | 9 |
| Zivadinov et al., 2018 | 1 | 1 | 1 | 0 | 1 | 1 | 1 | 0 | 0 | 1 | 0 | 0 | 0 | 0 | 0 | 1 | 0 | 1 | 0 | 1 | 0 | 0 | 0 | 0 | 0 | 0 | - | 10 |

Note: Power was not rated due to lack of clarity about the power criteria for the Downs and Black checklist.

# **Appendix: Downs and Black checklist for the assessment of the methodological quality of both randomized and non-randomized studies.**

**Reporting (1-10)**

1. Is the hypothesis/aim/objective of the study clearly described?

2. Are the main outcomes to be measured clearly described in the Introduction or Methods section?

3. Are the characteristics of the patients included in the study clearly described?

4. Are the interventions of interest clearly described?

5. Are the distributions of principal confounders in each group of subjects to be compared clearly described?

6. Are the main findings of the study clearly described?

7. Does the study provide estimates of the random variability in the data for the main outcomes?

8. Have all important adverse events that may be a consequence of the intervention been reported?

9. Have the characteristics of patients lost to follow-up been described?

10. Have the probability values been reported (e.g., 0.035 rather than <0.05) for the main outcomes except where the probability value is less than 0.001?

**External Validity (11-13)**

11. Were the subjects asked to participate in the study representative of the entire population from which they were recruited?

12. Were those subjects who were prepared to participate representative of the entire population from which they were recruited?

13. Were the staff, places, and facilities where the patients were treated, representative of the treatment the majority of patients receive?

**Internal Validity-Bias (14-20)**

14. Was an attempt made to blind study subjects to the intervention they have received?

15. Was an attempt made to blind those measuring the main outcomes of the intervention?

16. If any of the results of the study were based on “data dredging”, was this made clear?

17. In trials and cohort studies, do the analyses adjust for different lengths of follow-up of patients, or in case-control studies, is the time-period between the intervention and outcome the same for cases and controls?

18. Were the statistical tests used to assess the main outcomes appropriate?

19. Was compliance with the intervention/s reliable?

20. Were the main outcome measures used accurate (valid and reliable)?

**Internal Validity Confounding-Selection Bias**

21. Were the patients in different intervention groups (trials and cohort studies) or were the cases and controls (case-control studies) recruited from the same population?

22. Were study subjects in different intervention groups (trials and cohort studies) or were the cases and controls (case-control studies) recruited over the same period of time?

23. Were the study subjects randomised to intervention groups?

24. Was the randomised intervention assignment concealed from both patients and health are staff until recruitment was complete and irrevocable?

25. Was there adequate adjustment for confounding in the statistical analyses from which the main findings were drawn?

26. Were losses of patients to follow-up taken into account?

**Power**

27. Did the study have sufficient power to detect a clinically important effect where the probability value for a difference being due to chance is less than 5%?

# References

Bryant, B. R., Narapareddy, B. R., Bray, M. J. C., Richey, L. N., Krieg, A., Shan, G., … Bernick, C. B. (2020). The effect of age of first exposure to competitive fighting on cognitive and other neuropsychiatric symptoms and brain volume. *International Review of Psychiatry*, *32*(1), 89–95. https://doi.org/http://dx.doi.org/10.1080/09540261.2019.1665501

Casson, I., Viano, D., Haacke, E. M., Kou, Z., & LeStrange, D. G. (2014). Is there chronic brain damage in retired NFL players? Neuroradiology, neuropsychology, and neurology examinations of 45 retired players. *Sports Health*, *6*(5), 384–395. https://doi.org/https://dx.doi.org/10.1177/1941738114540270

Chong, C., Peplinski, J. S., Ross, K. B., & Berisha, V. (2018). Differences in fibertract profiles between patients with migraine and those with persistent post-traumatic headache. *Headache*, *58*(8), 1290–1291. https://doi.org/http://dx.doi.org/10.1111/head.13411

Clark, M. D., Varangis, E. M. L., Champagne, A. A., Giovanello, K. S., Shi, F., Kerr, Z. Y., … Guskiewicz, K. M. (2018). Effects of career duration, concussion history, and playing position on white matter microstructure and functional neural recruitment in former college and professional football athletes. *Radiology*, *286*(3), 967–977. https://doi.org/https://dx.doi.org/10.1148/radiol.2017170539

De Beaumont, L., Tremblay, S. S., Henry, L. C., Poirier, J., Lassonde, M., Théoret, H., & Theoret, S. (2013). Motor system alterations in retired former athletes: The role of aging and concussion history. *BMC Neurology*, *13*(1), 109. https://doi.org/http://dx.doi.org/10.1186/1471-2377-13-109

Ford, J. H., Giovanello, K. S., & Guskiewicz, K. M. (2013). Episodic memory in former professional football players with a history of concussion: An event-related functional neuroimaging study. *Journal of Neurotrauma*, *30*(20), 1683–1701. https://doi.org/10.1089/neu.2012.2535

Gardner, R. C., Hess, C. P., Brus-Ramer, M., Possin, K. L., Cohn-Sheehy, B. I., Kramer, J. H., … Miller, B. (2016). Cavum septum pellucidum in retired American pro-football players. *Journal of Neurotrauma*, *33*(1), 157–161. https://doi.org/http://dx.doi.org/10.1089/neu.2014.3805

Gilmore, C. S., Lim, K. O., Garvin, M. K., Wang, J.-K., Ledolter, J., Fenske, A. L., … Kardon, R. H. (2020). Association of optical coherence tomography with longitudinal neurodegeneration in veterans with chronic mild traumatic brain injury. *JAMA Network Open*, *3*(12), e2030824. https://doi.org/http://dx.doi.org/10.1001/jamanetworkopen.2020.30824

Goswami, R., Dufort, P., Tartaglia, M. C., Green, R. E., Crawley, A., Tator, C. H., … Davis, K. D. (2016). Frontotemporal correlates of impulsivity and machine learning in retired professional athletes with a history of multiple concussions. *Brain Structure and Function*, *221*(4), 1911–1925. https://doi.org/10.1007/s00429-015-1012-0

Haglund, Y., & Bergstrand. (1990). Does Swedish amateur boxing lead to chronic brain damage? 2. A retrospective study with CT and MRI. *Acta Neurologica Scandinavica*, *82*(5), 297–302. Retrieved from http://ovidsp.ovid.com/ovidweb.cgi?T=JS&PAGE=reference&D=emed4&NEWS=N&AN=21003567

Hampshire, A., MacDonald, A., & Owen, A. M. (2013). Hypoconnectivity and hyperfrontality in retired american football players. *Scientific Reports*, *3*, 1–8. https://doi.org/10.1038/srep02972

Hart, J. J., Kraut, M. A., Womack, K. B., Strain, J., Didehbani, N., Bartz, E., … Cullum, C. M. (2013). Neuroimaging of cognitive dysfunction and depression in aging retired National Football League players: a cross-sectional study. *JAMA Neurology*, *70*(3), 326–335. https://doi.org/https://dx.doi.org/10.1001/2013.jamaneurol.340

June, D., Williams, O. A., Huang, C.-W., An, Y., Landman, B. A., Davatzikos, C., … Beason-Held, L. L. (2020). Lasting consequences of concussion on the aging brain: Findings from the Baltimore Longitudinal Study of Aging. *NeuroImage*, *221*, 117182. https://doi.org/http://dx.doi.org/10.1016/j.neuroimage.2020.117182

Kelman, J. C., Hodge, C., Stanwell, P., Mustafic, N., & Fraser, C. L. (2020). Retinal nerve fibre changes in sports-related repetitive traumatic brain injury. *Clinical & Experimental Ophthalmology*, *48*(2), 204–211. https://doi.org/https://dx.doi.org/10.1111/ceo.13673

Koerte, I. K., Hufschmidt, J., Muehlmann, M., Tripodis, Y., Stamm, J. M., Pasternak, O., … Shenton, M. E. (2016). Cavum septi pellucidi in symptomatic former professional football players. *Journal of Neurotrauma*, *33*(4), 346–353. https://doi.org/10.1089/neu.2015.3880

Koerte, I. K., Lin, A. P., Muehlmann, M., Merugumala, S., Liao, H., Starr, T., … Shenton, M. E. (2015). Altered neurochemistry in former professional soccer players without a history of concussion. *Journal of Neurotrauma*, *32*(17), 1287–1293. https://doi.org/10.1089/neu.2014.3715

Koerte, I. K., Mayinger, M., Muehlmann, M., Kaufmann, D., Lin, A. P., Steffinger, D., … Shenton, M. E. (2016). Cortical thinning in former professional soccer players. *Brain Imaging and Behavior*, *10*(3), 792–798. https://doi.org/https://dx.doi.org/10.1007/s11682-015-9442-0

Kuhn, A. W., Zuckerman, S. L., Solomon, G. S., Casson, I. R., & Viano, D. C. (2017). Interrelationships among neuroimaging biomarkers, neuropsychological test data, and symptom reporting in a cohort of retired National Football League players. *Sports Health*, *9*(1), 30–40. https://doi.org/http://dx.doi.org/10.1177/1941738116674006

Lepage, C., Muehlmann, M., Tripodis, Y., Hufschmidt, J., Stamm, J., Green, K., … Koerte, I. K. (2019). Limbic system structure volumes and associated neurocognitive functioning in former NFL players. *Brain Imaging and Behavior*, *13*(3), 725–734. https://doi.org/http://dx.doi.org/10.1007/s11682-018-9895-z

Lin, A. P., Ramadan, S., Stern, R. A., Box, H. C., Nowinski, C. J., Ross, B. D., & Mountford, C. E. (2015). Changes in the neurochemistry of athletes with repetitive brain trauma: Preliminary results using localized correlated spectroscopy. *Alzheimer’s Research and Therapy*, *7*(1), 1–9. https://doi.org/10.1186/s13195-015-0094-5

Lipton, M. L., Kim, N., Zimmerman, M. E., Kim, M., Stewart, W. F., Branch, C. A., & Lipton, R. B. (2013). Soccer heading is associated with white matter microstructural and cognitive abnormalities. *Radiology*, *268*(3), 850–857. https://doi.org/10.1148/radiol.13130545

McCrory, P., Meeuwisse, W., Johnston, K., Dvorak, J., Aubry, M., Molloy, M. ., & Cantu, R. (2009). Consensus Statement on Concussion in Sport – the 3rd International Conference on Concussion in Sport held in Zurich, november 2008. *South African Journal of Sports Medicine*, *21*(2).

McCrory, Paul, Meeuwisse, W. H., Aubry, M., Cantu, R. C., Dvorák, J., Echemendia, R. J., … Turner, M. (2013). Consensus Statement on Concussion in Sport-The 4th International Conference on Concussion in Sport Held in Zurich, November 2012. *PM and R*, *5*(4), 255–279. https://doi.org/10.1016/j.pmrj.2013.02.012

Meeuwisse, W. H., Schneider, K. J., Dvořák, J., Omu, O. T., Finch, C. F., Hayden, K. A., & McCrory, P. (2017). The Berlin 2016 process: a summary of methodology for the 5th International Consensus Conference on Concussion in Sport. *British Journal of Sports Medicine*, *51*(11), 873–876. https://doi.org/10.1136/bjsports-2017-097569

Menon, D. K., Schwab, K., Wright, D. W., & Maas, A. I. (2010, November 1). Position statement: Definition of traumatic brain injury. *Archives of Physical Medicine and Rehabilitation*. W.B. Saunders. https://doi.org/10.1016/j.apmr.2010.05.017

Mild Traumatic Brain Injury Committee. (1993). American Congress of Rehabilitation Medicine, Head Injury Interdisciplinary Special Interest Group: Definition of mild traumatic brain injury. *The Journal of Head Trauma Rehabilitation*, *8*(3), 86–87.

Misquitta, K., Dadar, M., Tarazi, A., Hussain, M. W., Alatwi, M. K., Ebraheem, A., … Tartaglia, M. C. (2018). The relationship between brain atrophy and cognitive-behavioural symptoms in retired Canadian football players with multiple concussions. *NeuroImage: Clinical*, *19*, 551–558. https://doi.org/http://dx.doi.org/10.1016/j.nicl.2018.05.014

Monti, J. M., Voss, M. W., Pence, A., McAuley, E., Kramer, A. F., & Cohen, N. J. (2013). History of mild traumatic brain injury is associated with deficits in relational memory, reduced hippocampal volume, and less neural activity later in life. *Frontiers in Aging Neuroscience*, *5*(AUG), 1–9. https://doi.org/10.3389/fnagi.2013.00041

Multani, N., Goswami, R., Khodadadi, M., Ebraheem, A., Davis, K. D., Tator, C. H., … Tartaglia, M. C. (2016). The association between white-matter tract abnormalities, and neuropsychiatric and cognitive symptoms in retired professional football players with multiple concussions. *Journal of Neurology*, *263*(7), 1332–1341. https://doi.org/https://dx.doi.org/10.1007/s00415-016-8141-0

Qualifying Statements. (2009). VA/DoD clinical practice guideline for management of concussion/mild traumatic brain injury. *Journal of Rehabilitation Research & Development*, *46*(6), 1–60.

Rajesh, A., Cooke, G. E., Monti, J. M., Jahn, A., Daugherty, A. M., Cohen, N. J., & Kramer, A. F. (2017). Differences in brain architecture in remote mild traumatic brain injury. *Journal of Neurotrauma*, *34*(23), 3280–3287. https://doi.org/http://dx.doi.org/10.1089/neu.2017.5047

Robbins, C., Daneshvar, D., Picano, J., Gavett, B., Baugh, C., Riley, D., … McKee, A. (2014). Self-reported concussion history: impact of providing a definition of concussion. *Open Access Journal of Sports Medicine*, *5*, 99. https://doi.org/10.2147/oajsm.s58005

Rowland, J. A., Stapleton-Kotloski, J. R., Dobbins, D. L., Rogers, E., Godwin, D. W., & Taber, K. H. (2018). Increased small-world network topology following deployment-acquired traumatic brain injury associated with the development of post-traumatic stress disorder. *Brain Connectivity*, *8*(4), 205–211. https://doi.org/https://dx.doi.org/10.1089/brain.2017.0556

Small, G. W., Kepe, V., Siddarth, P., Ercoli, L. M., Merrill, D. A., Donoghue, N., … Barrio, J. R. (2013). PET scanning of brain tau in retired national football league players: Preliminary findings. *American Journal of Geriatric Psychiatry*, *21*(2), 138–144. https://doi.org/https://doi.org/10.1016/j.jagp.2012.11.019

Tator, C. H. (2013). Concussions and their consequences: Current diagnosis, management and prevention. *CMAJ*, *185*(11), 975–979. https://doi.org/10.1503/cmaj.120039

Tremblay, S., De Beaumont, L., Henry, L. C., Boulanger, Y., Evans, A. C., Bourgouin, P., … Lassonde, M. (2013). Sports concussions and aging: A neuroimaging investigation. *Cerebral Cortex*, *23*(5), 1159–1166. https://doi.org/http://dx.doi.org/10.1093/cercor/bhs102

Tremblay, S., Desjardins, M., Bermudez, P., Iturria-Medina, Y., Evans, A. C., Jolicoeur, P., & De Beaumont, L. (2019). Mild traumatic brain injury: The effect of age at trauma onset on brain structure integrity. *NeuroImage. Clinical*, *23*, 101907. https://doi.org/http://dx.doi.org/10.1016/j.nicl.2019.101907

Vasilevskaya, A., Taghdiri, F., Burke, C., Tarazi, A., Naeimi, S. A., Khodadadi, M., … Tartaglia, M. C. (2020). Interaction of APOE4 alleles and PET tau imaging in former contact sport athletes. *NeuroImage: Clinical*, *26*, 102212. https://doi.org/http://dx.doi.org/10.1016/j.nicl.2020.102212

Wang, M.-L., Wei, X.-E., Yu, M.-M., Li, P.-Y., & Li, W.-B. (2017). Self-reported traumatic brain injury and in vivo measure of AD-vulnerable cortical thickness and AD-related biomarkers in the ADNI cohort. *Neuroscience Letters*, *655*, 115–120. https://doi.org/https://dx.doi.org/10.1016/j.neulet.2017.06.055

Ware, A. L., Wilde, E. A., Newsome, M. R., Moretti, P., Abildskov, T., Vogt, G. S., … Levin, H. S. (2020). A preliminary investigation of corpus callosum subregion white matter vulnerability and relation to chronic outcome in boxers. *Brain Imaging and Behavior*, *14*(3), 772–786. https://doi.org/http://dx.doi.org/10.1007/s11682-018-0018-7

Wilde, E. A., Hunter, J. V., Li, X., Amador, C., Hanten, G., Newsome, M. R., … Levin, H. S. (2016). Chronic effects of boxing: Diffusion tensor imaging and cognitive findings. *Journal of Neurotrauma*, *33*(7), 672–680. https://doi.org/10.1089/neu.2015.4035

Zivadinov, R., Polak, P., Schweser, F., Bergsland, N., Hagemeier, J., Dwyer, M. G., … Willer, B. S. (2018). Multimodal imaging of retired professional contact sport athletes does not provide evidence of structural and functional brain damage. *Journal of Head Trauma Rehabilitation*, *33*(5), E24–E32. https://doi.org/10.1097/HTR.0000000000000422
